# Arbutin alleviates fatty liver by inhibiting ferroptosis via FTO/SLC7A11 pathway

**DOI:** 10.1016/j.redox.2023.102963

**Published:** 2023-11-16

**Authors:** Tianyu Jiang, Yao Xiao, Jinfeng Zhou, Zupeng Luo, Lin Yu, Qichao Liao, Siqi Liu, Xinyi Qi, Hao Zhang, Menglong Hou, WeiWei Miao, Batbold Batsaikhan, Turtushikh Damba, Yunxiao Liang, Yixing Li, Lei Zhou

**Affiliations:** a Institute of Digestive Disease, Guangxi Academy of Medical Sciences, The People's Hospital of Guangxi Zhuang Autonomous Region, Nanning, 530021, China; b College of Animal Science and Technology, Guangxi University, Nanning, 530004, China; c Department of Internal Medicine, Institute of Medical Sciences, Mongolian National University of Medical Sciences, Ulaanbaatar, Mongolia; d Department of Health Research, Graduate School, Mongolian National University of Medical Sciences, Ulaanbaatar, Mongolia; e School of Pharmacy, Mongolian National University of Medical Sciences, Ulaanbaatar, Mongolia

**Keywords:** Arbtuin, Non-alcoholic fatty liver disease, Ferroptosis, FTO, m6A methylation modification, SLC7A11

## Abstract

Non-alcoholic fatty liver disease (NAFLD) is a potentially serious disease that affects 30 % of the global population and poses a significant risk to human health. However, to date, no safe, effective and appropriate treatment modalities are available. In recent years, ferroptosis has emerged as a significant mode of cell death and has been found to play a key regulatory role in the development of NAFLD. In this study, we found that arbutin (ARB), a natural antioxidant derived from *Arctostaphylos uva-ursi (L.)*, inhibits the onset of ferroptosis and ameliorates high-fat diet-induced NAFLD *in vivo* and *in vitro*. Using reverse docking, we identified the demethylase fat mass and obesity-related protein (FTO) as a potential target of ARB. Subsequent mechanistic studies revealed that ARB plays a role in controlling methylation of the *SLC7A11* gene through inhibition of FTO. In addition, we demonstrated that SLC7A11 could alleviate the development of NAFLD *in vivo* and *in vitro*. Our findings identify the FTO/SLC7A11 axis as a potential therapeutic target for the treatment of NAFLD. Specifically, we show that ARB alleviates NAFLD by acting on the FTO/SLC7A11 pathway to inhibit ferroptosis.

## Highlights

• Discovery of Arbutin effect on NAFLD.
• Arbutin mitigating effect on NAFLD is produced by ferroptosis.
• Discovery of the regulatory role of SLC7A11 in NAFLD.
• The FTO/SLC7A11 axis as a potential therapeutic target for the treatment of NAFLD.

## Graphical abstract

**Figure:**
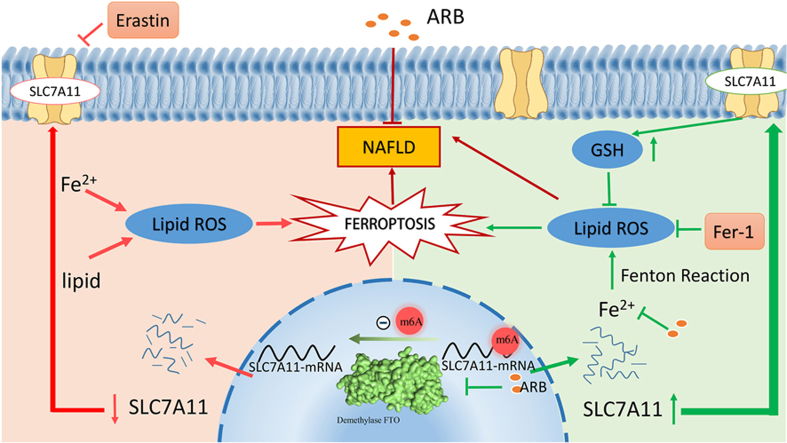

## 1 Introduction

Non-alcoholic fatty liver disease (NAFLD) is a potentially serious disease that affects 30 % of the world's population and has significant global implications [1]. The primary cause of NAFLD is the abnormal accumulation of fat in the liver, resulting in liver steatosis. This condition is often accompanied by metabolic abnormalities, including obesity, diabetes mellitus, and hyperlipidemia, which may act together to contribute to further disease progression [2,3]. Despite the increasing prevalence and global health impact of NAFLD, there remains a lack of approved therapeutic strategies due to an incomplete understanding of its pathogenesis.

In recent years, extensive research has shed light on the significant role of ferroptosis in the pathogenesis of liver diseases [4,5]. Ferroptosis is a unique type of oxidative cell death that is iron-dependent and associated with lipid peroxidation [6]. Ferroptosis is characterized by the iron-mediated Fenton reaction, which amplifies the generation of free radicals and reactive oxygen species (ROS). Intracellular iron overload and uncontrolled lipid peroxidation are two critical events that lead to ferroptosis [7]. Studies have shown a correlation between the development of NAFLD and deposition of iron in the liver [[8], [9], [10]]. Accumulation of iron in the liver can lead to oxidative stress and mitochondrial damage within hepatocytes. Indeed, reducing liver iron deposition using iron chelators has been shown to be beneficial in effectively alleviating symptoms and slowing the progression of NAFLD [11]. Since the liver plays a crucial role in iron storage and lipid metabolism, it is possible that inhibiting ferroptosis could emerge as a potential novel therapeutic strategy for the treatment of liver diseases.

The solute carrier family 7 member 11 (SLC7A11) gene, a component of system xc-, is responsible for the maintenance of intracellular glutathione (GSH) levels [12], which are closely associated with ferroptosis. The SLC7A11 gene has been reported to be transcriptionally regulated by two transcription factors: Nuclear factor erythroid 2-related factor 2 (Nrf2) and Nuclear factor erythroid 2-related factor 1 (Nrf1). In homeostatic conditions, Nrf1 effectively inhibits the non-specific *trans*-activation of SLC7A11. When the cell encounters oxidative stress, Nrf1 is displaced from the antioxidant response element (ARE) in the gene promoter, and Nrf2 is recruited to the ARE to *trans*-activate the transcription of SLC7A11 [13]. In addition to the transcriptional regulation of Nrf1 and Nrf2, it has been observed in thyroid carcinoma (PTC) tissues that demethylases, such as the demethylase fat mass and obesity-associated protein (FTO), can promote ferroptosis by down-regulating m6A methylation of SLC7A11 [14]. However, the role and regulatory mechanisms of action of the FTO/SLC7A11 pathway in NAFLD have not been extensively studied. Therefore, exploring the role and regulatory mechanisms of this pathway in the liver will greatly contribute to our understanding of the pathogenesis of NAFLD and provide new insights for the development of therapeutic strategies.

Arbtuin (ARB) is a glucoside derivative of hydroquinone that exhibits various pharmacological activities, including antioxidant, anti-inflammatory, and antibacterial properties, and is commonly used in cosmetics and herbal dietary supplements [15]. Several studies have demonstrated the favorable outcomes of ARB in preventing obesity, alcoholic fatty liver disease (ALD), and liver injury. Previous studies have indicated that ARB exhibits hepatoprotective effects against liver injury induced by α-Naphthylisothiocyanate [16]. In addition, ARB has been shown to alleviate ALD through the modulation of oxidative stress and the Nrf-2/HO-1 signaling pathway [17]. Moreover, ARB may be a potential regulator of lipid metabolism, since it has been shown to affect adipocyte differentiation and lipid accumulation in 3T3-L1 adipocytes [18]. Although ARB has been shown to modulate oxidative stress and lipid metabolism, its specific mechanism of action in NAFLD, particularly its potential involvement in ferroptosis, remains unknown.

In the present study, we examined the potential alleviating effects of ARB on NAFLD and elucidated the role of ferroptosis in the pathogenesis of NAFLD. In addition, our study is the first to demonstrate the significance of SLC7A11 in ameliorating NAFLD, which is regulated through FTO. We identified the ARB/FTO/SLC7A11 pathway as a novel approach for the prevention and treatment of fatty liver.

## 2 Materials and methods

### 2.1 Animals and experimental design

The animal experiments in this study complied with animal care laws and guidelines. All procedures were approved by the Guangxi University Laboratory Animal Ethics Committee (GXU-2023-0094). A total of 43 male San C57BL/6 mice (SPF Bio-Tech Co., Ltd., Beijing, China), aged six weeks, were obtained from a commercial hatchery. The mice were housed under standard conditions, with a temperature of 25 ± 2 °C and a relative humidity of 50 ± 5 %. A 12-h on/off lighting cycle was implemented using incandescent lamps. The mice were provided with ad libitum access to feed and water throughout the study.

To investigate the effects of arbutin on high-fat diet-induced obesity, randomly assigned mice were divided into three groups (n = 6 per group): (1) Control group, fed a basic diet; (2) HFD group, fed a high-fat diet; (3) HFD + ARB group, fed with the addition of 1g of ARB per kg of high-fat diet.

The part of the mouses were randomly divided into four groups (n = 6–7 per group). (1) Control group: tail vein injection of control viral fluid and feed basic diet; (2) CON-SLC7A11 group: tail vein injection of SLC7A11 viral fluid and feed basic diet; (3) HFD group: tail vein injection of control viral fluid and feed high-fat diet; (4) HFD-SLC7A11 group: tail vein injection of SLC7A11 viral fluid and feed high-fat diet. The basic diet (TP2330055MC, 3.8 kcal/g, 10 % fat calories, 14 % protein calories, 76 % carbohydrate calories), The high-fat diet (TP2330055 M, Calories: 5.5 kcal/g, 60 % fat calories, 14 % protein calories, 26 % carbohydrate calories) and ARB customized feeds were sourced from Trophic Bio-Tech Co., Ltd. Nantong, China.

### 2.2 Nuclear magnetic resonance (NMR)

Using nuclear magnetic resonance (NMR) technology, (Niumag QMR23-060H–I instrument Suzhou, China), the fat and lean meat rates of mice were measured over eight weeks. Prior to each measurement, the machine was calibrated and the body weight of the mice was entered into the system. During the test, the mice were placed into the instrument and the test was initiated. This testing process was repeated once a week for a period of eight weeks.

### 2.3 Metabolic cage

At least 3 mice are randomly selected from each group for metabolic cage (Promethion Cages, Sable Systems International, Las Vegas, Nevada, U.S.A.) experiments. Metabolic cages recorded exercise, O_2_ consumption, CO_2_ production, respiratory exchange rate (RER), and energy expenditure at 5 min intervals for 48 h as previously described [19].

### 2.4 Micro-CT

After anesthetizing mice with tribromoethanol, scanned the mice using Micro-CT (SkyScan 1278, Bruker, Billerica, Massachusetts, U.S.A.) to detect the body fat percentage.

### 2.5 Cells and cells cultures

HepG2 cells cultured in Dulbecco's modified Eagle medium (DMEM) supplemented with 10 % fetal bovine serum (FBS) and 1 % antibiotics (penicillin-streptomycin liquid) at 37 °C in a 5 % CO_2_ cell culture incubator. Arbutin (purity >99.96 %) was purchased from Yuanye Bio-Technology Co., Ltd. (Shanghai, China).

To culture HepG2 cells, DMEM supplemented with 10 % FBS was used to achieve 80 % confluency, followed by a 6-h resting period in serum-free DMEM. Cells were then treated with OA/PA DMEM (OA:200 μM PA:100 μM) containing ARB (0–100 μM) for 24 h.

### 2.6 Cell proliferation and cytotoxicity assay

After the cell seeding and 96-well plate growth to 80 % confluency, treat with 0, 10, 25, 50, 75, 100 μM arbutin for 24h. Using the CCK-8 assay kit (Yeasen Biotechnology Co., Ltd. Shanghai, China) cell viability is detected after 1 h of co-incubation with cells.

### 2.7 Tissue/cell TG、TC assays

Tissue samples from mice were lysed with anhydrous ethanol and crush using a tissue crusher. Subsequently tested according to the manufacturer's method and corrected for the weight of the liver. Cell samples were centrifuged with RIPA lysate containing 1 % PMSF at 4 °C, subjected to lysis for 30 min. Subsequently tested according to the manufacturer's method and detected protein concentrations for normalization. TG and TC values of the cells and tissue samples were assessed by the TG and TC kits (Jiancheng Co., Ltd. Nanjing, China).

### 2.8 Tissue and cell iron ion/ferrous ion measure

According to the manufacturer's instructions, tissue and cell iron or ferrous ion concentrations used iron ion assay kits and ferrous ion assay kits (Elabscience Biotechnology Co.,Ltd, Wuhan, China). Results were normalized to the number of cells.

### 2.9 Mitochondrial ROS test

Cellular mitochondrial ROS content was assayed using MitoSOX (Thermo Fisher, Waltham, MA). After cell treatment, 5 μM MitoSOX was used to co-incubate with the cells for 10 min at 37 °C, and the cells were washed twice using PBS before observing the cell fluorescence intensity under an inverted microscope (IX53; Olympus Corporation, Tokyo, Japan) and fluorescence quantification was measured using microplate reader, and the proteins were detected using the BCA method for normalization.

### 2.10 Tissue ROS and MDA assay

The ROS and MDA levels in the liver were assessed using Mice ROS ELISA KIT (Enzyme-linked Biotechnology Co., Ltd. Shanghai, China) and MDA ELISA Kit (Elabscience Biotechnology Co., Ltd, Wuhan, China). Liver tissue was homogenized using PBS (Phosphate-Buffered Saline), centrifuged and the supernatant was taken and assayed according to the manufacturer's instructions, and the proteins were detected using the BCA method for normalization.

### 2.11 Cellular thermal shift assay (CETSA)

Cells were extracted using M-PER (Solarbio Science & Technology Co., Ltd. Beijing, China), and the cell supernatant was divided into three groups after centrifugation. Two groups were incubated with the same concentration of DMSO (Solarbio Science & Technology Co., Ltd. Beijing, China) and ARB at 37 °C for 2 h, and then divided into PCR tubes. The protein was denatured using a PCR instrument at the corresponding temperature for 3 min. After centrifuging at 12,000 g at 4 °C for 20 min, the supernatant was mixed with loading buffer in a ratio of 3:1 and boiled at 100 °C. The obtained samples were subjected to Western Blot.

### 2.12 m6A dot blot assay

After total RNA extraction, the samples were diluted to 400, 200, and 100 ng. mRNA samples were denatured at 95 °C for 3 min, and then incubated on ice for 5 min. The samples were then loaded onto a nitrocellulose filter membrane (Solarbio Science & Technology Co., Ltd. Beijing, China) along with 20 × SSC buffer (Beyotime Biotechnology, Beijing, Cnina). The membrane was crosslinked under UV light for 5 min and washed with PBST three times. After blocking with 5 % non-fat milk, the membrane was incubated with a specific m6A antibody at 4 °C overnight. The membrane was then incubated with HRP-conjugated anti-mouse IgG for 1 h and observed using the BIO-RAD Gel Doc XR system.

### 2.13 MeRIP-seq and MeRIP-qPCR

MeRIP-seq results from cells overexpressing or suppressing FTO from others study were used to analyze the m6A methylation site of SLC7A11 (GEO accession: GSE154561, GSE189465). The MeRIP procedure was performed according to the instructions provided by the manufacturer using a MeRIP™ m6A Transcriptome Profiling Kit (RIBOBIO ribo, Guangzhou, China). First, total RNA was extracted from the cells and fragmented by incubation with a 94 °C PCR machine for 3 min. The fragmented RNA was then precipitated overnight with sodium acetate and glycogen at −20 °C, and the resulting precipitate was collected by ethanol extraction. Anti m6A magnetic beads were prepared from Magnetic beads A/G and m6A antibodies, and the RNA was immunoprecipitated with the supernatant of the precipitation products using these beads. After full washing, the RNA was recovered, and subsequent q-PCR detection was performed to analyze the m6A methylation site of SLC7A11.

### 2.14 Statistical analysis

The data between two groups were analyzed by independent samples *t*-test, and one-way ANOVA was used to compare the means of three groups. Data were presented as means ± SEMs from three independent experiments. The differences were considered statistically significant if (∗) p < 0.05 or (∗∗) p < 0.01.

### 2.15 Other methods

Detailed experimental protocols and additional assays are described in the Supplementary Material.

## 3 Results

### 3.1 ARB regulates intracellular lipid deposition

**Fig. 1:**
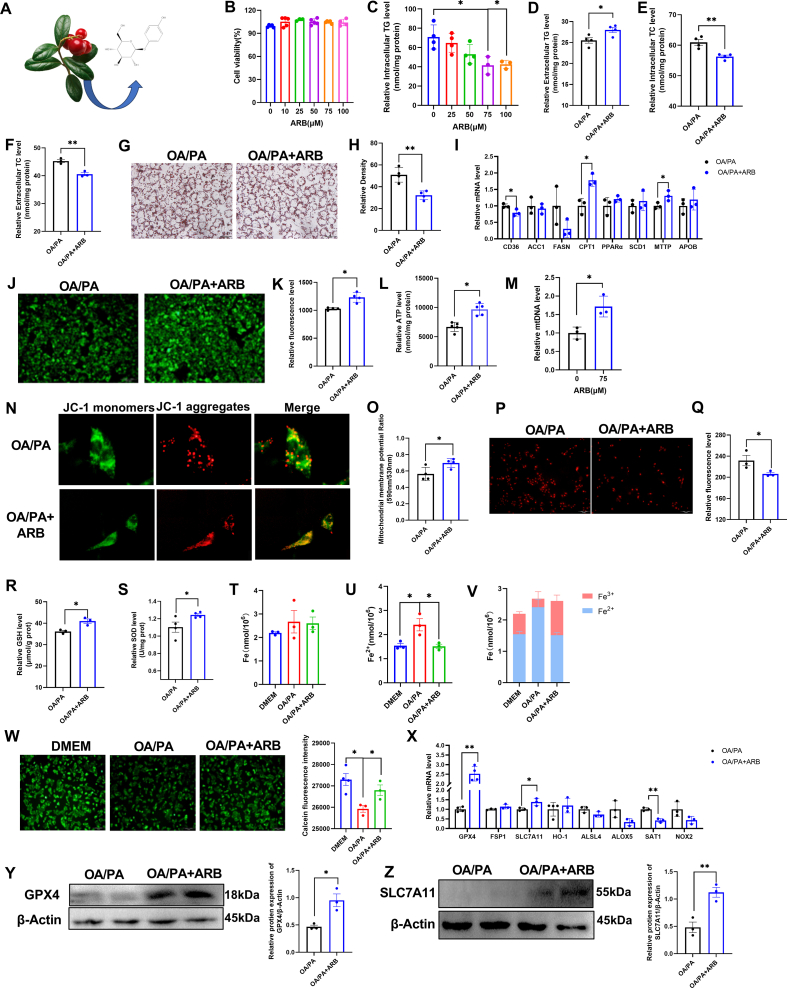
**ARB regulates intracellular lipid deposition and ferroptosis.** (A) Molecular structure of ARB. (B) CCK8 detects the effect of ARB on cell viability (n = 4–5 per group). (C–D) Intracellular and extracellular TG content under different concentrations of ARB treatment for 24 h (C: n = 3–4 per group, D: n = 4 per group). (E–F) Intracellular and extracellular TC content under different concentrations of ARB treatment for 24 h (E: n = 4 per group, F: n = 3 per group). (G–H) Oil red O staining and its quantification (n = 4 per group). (I) qPT-PCR of lipid metabolism-related genes (n = 3 per group). (J–K) Mitochondrial staining and its quantification (n = 4 per group). (L) Effect of 75 μM ARB on intracellular ATP levels (n = 5 per group). (M) Effect of 75 μM ARB on mtDNA (n = 3 per group). (N–O) Mitochondrial membrane potential and its quantification (n = 4 per group). (P–Q) ROS staining with Mitosox red probe and its quantification (n = 3 per group). (R–S) Effect of 75 μM ARB on intracellular GSH and SOD content (R: n = 3 per group, S: n = 4 per group). (T–V) Effect of 75 μM ARB on intracellular Fe, Fe^2+^ and the ratio of Fe^2+^ to Fe^3+^ (n = 3 per group). (W) Calcein staining and its quantification (n = 3–4 per group). (X) qPT-PCR of ferroptosis-related genes (n = 3–4 per group). (Y–Z) Western Blot detects the protein expression levels of GPX4 and SLC7A11 under ARB treatment (n = 3 per group). Data are mean ± SEM, *n* ≥ 3; The data between two groups were analyzed by independent samples *t*-test, and one-way ANOVA was used to compare the means of three groups, ∗*P* < 0.05; ∗∗*P* < 0.01. (For interpretation of the references to colour in this figure legend, the reader is referred to the Web version of this article.)

ARB is derived from the leaves of *Arctostaphylos uva-ursi (L.)* and its molecular formula is shown in Fig. 1A. Here, we established a cellular model of lipid deposition by treating HepG2 cells with oleic acid and palmitic acid (OA/PA). First, we used CCK-8 and scratch assays to examine the effects of ARB on cell viability. We found that ARB had no effect on HepG2 cell viability at concentrations of up to 100 μM (Fig. 1B, Figs. S1A–B). ARB was found to reduce intracellular TG levels in HepG2 cells, with a more pronounced effect at 75 μM (Fig. 1C). Similarly, ARB had no effect on cell viability and reduced intracellular TG levels in 3T3-L1 cells (Figs. S1C–D). Based on these results, we used ARB at a concentration of 75 μM in subsequent experiments. ARB was found to increase extracellular TG levels and significantly reduce both intracellular and extracellular TC levels (Fig. 1D–F). Oil red O staining revealed that ARB treatment of OA/PA-treated HepG2 cells led to a significant reduction in intracellular lipid droplet accumulation compared to OA/PA-treated cells (Fig. 1G and H).

Next, we quantified the expression levels of genes associated with lipid metabolism in HepG2 cells. We found that ARB treatment inhibited the expression of lipid synthesis genes, such as CD36 and FANS, but promoted expression of genes involved in lipid catabolism and lipid transport, such as CPT1 and MTTP (Fig. 1I). Together, these findings suggested that ARB reduces intracellular lipid accumulation without affecting cell viability.

### 3.2 ARB inhibits ferroptosis by regulating cellular energy metabolism and oxidative stress

Since the development of NAFLD has been closely associated with metabolic dysregulation and oxidative stress, we next sought to determine whether ARB affects energy metabolism and oxidative stress. We found that ARB significantly increased the number of mitochondria, as well as ATP levels in OA/PA-treated cells (Fig. 1J-M). In addition, our mitochondrial membrane potential (MMP) assay revealed a significant increase in MMP levels following ARB treatment of OA/PA-treated cells (Fig. 1N-O). At the same time, ARB treatment led to an increase in intracellular GSH, superoxide dismutase (SOD), and catalase (CAT) levels, and a reduction in Tatol ROS, mitochondrial ROS and malondialdehyde (MDA) production (Fig. 1P–S, Figs. S2A–C).

We further examined the effects of ARB on intracellular levels of total iron (Fe) and ferrous ions (Fe^2+^) in OA/PA-treated cells, and found that ARB treatment significantly reduced the intracellular Fe^2+^ content (Fig. 1T–V). Furthermore, calcein staining revealed that ARB treatment of OA/PA-treated HepG2 cells reversed the increase in unstable intracellular iron pools observed after OA/PA treatment alone (Fig. 1W).

We found that ARB treatment significantly up-regulated the expression of ferroptosis-suppressor genes, such as GPX4 and SLC7A11, while significantly down-regulating the expression of the ferroptosis-promoting gene SAT1 (Fig. 1X). In addition, ARB treatment was found to significantly reduce hepcidin mRNA expression levels (Fig. S2D). Western blot analysis showed that ARB treatment of OA/PA-treated cells significantly increased GPX4 and SLC7A11 protein expression levels (Fig. 1Y-Z). Taken together, our results indicated that ARB may inhibit cellular ferroptosis in an OA/PA cellular model of NAFLD.

### 3.3 ARB reduces lipid deposition in mice

**Fig. 2:**
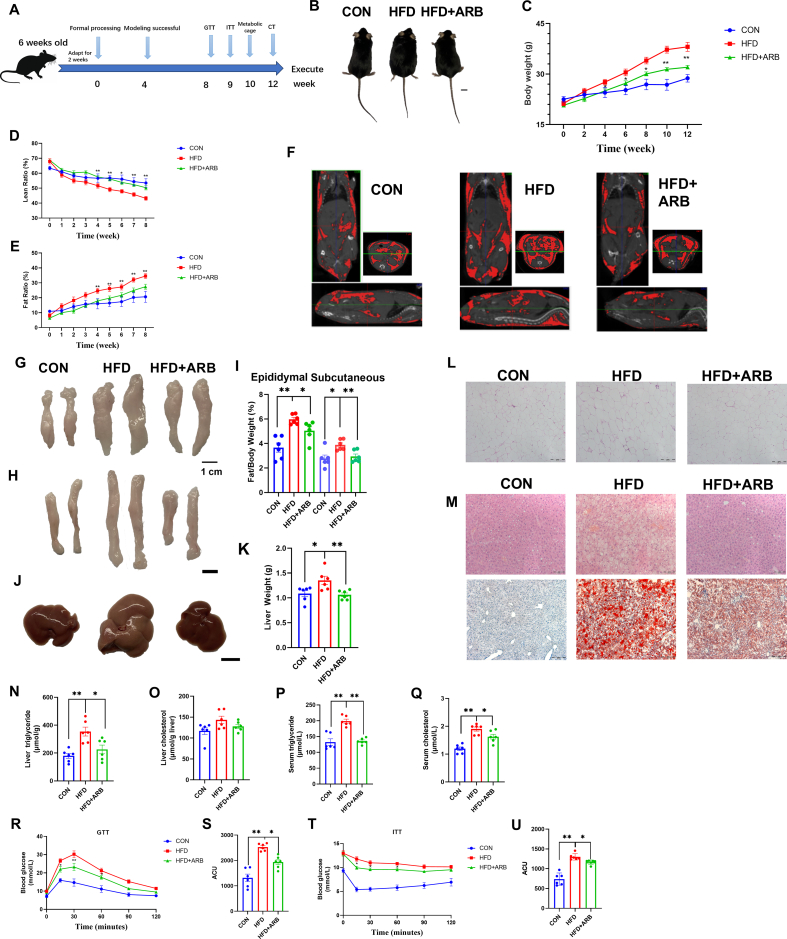
**ARB reduces lipid deposition in mice.** (A) Schematic diagram of the experimental process in mice. (B) Pictures of the body size of mice. (C–E) Body weight and body fat percentage of mice (n = 6 per group). (F) CT images. (G–I) Pictures of epididymal and subcutaneous fat and relative weights of mice (n = 6 per group). (J–K) Pictures of mice liver and relative weights. (L) H&E staining of epididymal fat. (M) H&E staining of liver (top) and Oil red O staining (bottom). (N–O) TG and TC content of mice liver (n = 6 per group). (P–Q) Serum TG, TC content in mice (n = 6 per group). (R–S) GTT assay at the eighth week of treatment (n = 6 per group). (T–U) ITT assay at the 9 weeks of treatment (n = 6 per group). Data are mean ± SEM, *n* = 6; One-way ANOVA was used to compare the means of three groups, ∗*P* < 0.05; ∗∗*P* < 0.01. (For interpretation of the references to colour in this figure legend, the reader is referred to the Web version of this article.)

To determine the effects of ARB on lipid deposition in mice, high-fat diet (HFD)-fed mice were administered ARB at a dosage of 1 g/kg. Mice fed a normal diet were considered to be the CON group. An outline of the experimental procedure is shown in Fig. 2A.

We found that the HFD led to a significant increase in body weight (Fig. 2B and C) and fat ratio (Fig. 2E) and a reduction in lean ratio (Fig. 2D), while ARB treatment of HFD-fed mice significantly attenuated these effects. Mice in the HFD + ARB group displayed significantly less fat deposition than HFD mice as measured by micro-CT (Fig. 2F). The relative weights of epididymal and subcutaneous fat, as well as liver tissue, were significantly reduced in the HFD + ARB group compared to the HFD group (Fig. 2G–K). H&E and Oil red O staining showed that ARB alleviated the damage and reduced lipid accumulation in the liver, as well as reduced fat size and area of epididymal fat compared with the HFD group (Fig. 2L-M).

ARB treatment of HFD mice led to a reduction in liver TG and TC levels, and fecal TG content compared to HFD mice (Fig. 2N-O, Fig. S3A). In addition, ARB significantly attenuated the HFD-induced increase in serum TG, TC, LDL-H and AST, ALT levels and decrease in HDL-H levels (Fig. 2P-Q, Figs. S3B–E). Together, these results suggested that ARB could alleviate HFD-induced lipid deposition and liver injury.

To further assess the effects of ARB treatment on glucose metabolism in HFD mice, we carried out GTT and ITT tests at weeks 9 and 10 of ARB treatment. Our GTT results showed that ARB significantly reduced the increase in blood glucose levels that occurred after the glucose injection compared to HFD mice, as well as promoted the return of blood glucose levels to normal levels (Fig. 2R and S). Our ITT results were consistent with the GTT data (Fig. 2T-U). Together, our findings suggested that supplementing the diet with ARB could ameliorate HFD-induced insulin resistance.

### 3.4 ARB modulates energy metabolism and oxidative stress in mice to ameliorate ferroptosis

**Fig. 3:**
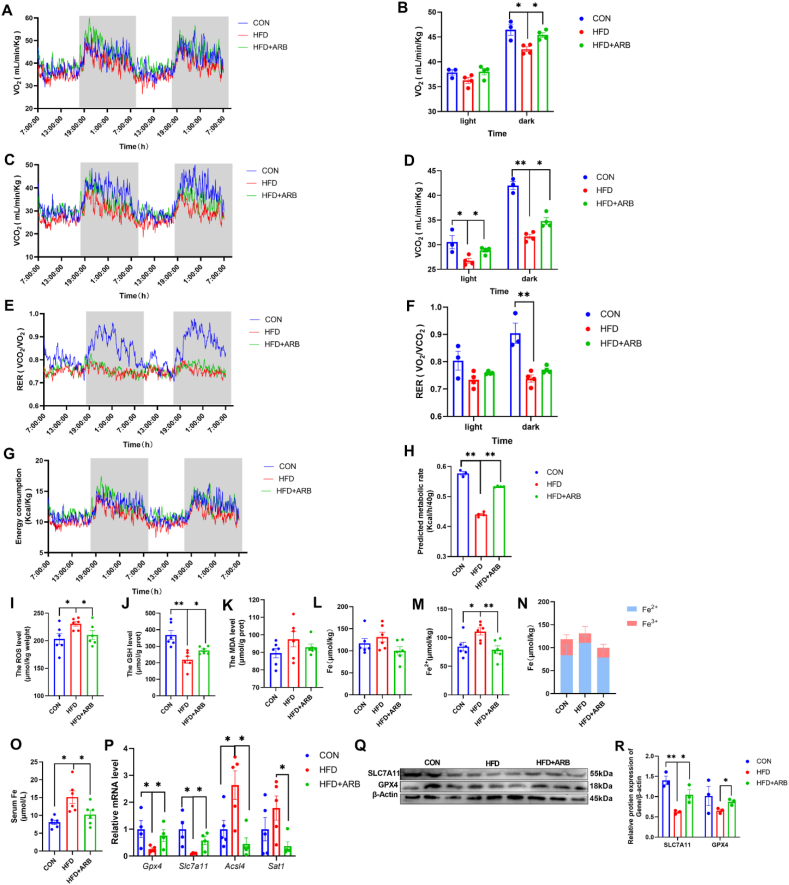
**ARB modulates energy metabolism and oxidative stress in mice to ameliorate ferroptosis.** (A–B) O_2_ consumed (n = 3–4 per group). (C–D) CO_2_ produced (n = 3–4 per group). (E–F) RER (VO_2_/VCO_2_) (n = 3–4 per group). (G–H) Energy metabolism (n = 3–4 per group). (I–K) Effect of 1 g/kg ARB on liver ROS, GSH, and MDA in mice (n = 6 per group). (L–N) Fe, Fe^2+^, and Fe^2+^ to Fe^3+^ ratio (n = 6 per group). (O) Serum Fe (n = 6 per group). (P) qPT-PCR of ferroptosis-related genes (n = 4–5 per group). (Q–R) Changes in SLC7A11 protein levels in mice liver of 1 g/kg ARB treatment (n = 6 per group). Data are mean ± SEM, *n* ≥ 3; ∗*P* < 0.05; ∗∗*P* < 0.01. (K–R) Data are mean ± SEM, *n* ≥ 6; One-way ANOVA was used to compare the means of three groups, ∗*P* < 0.05; ∗∗*P* < 0.01.

Following 10 weeks of ARB treatment, we performed a metabolic cage assay to measure energy expenditure and physical activity in the mice. We found significantly higher levels of CO_2_ production and O_2_ consumption in the HFD + ARB group compared to the HFD group (Fig. 3A–D). Similarly, the respiratory entropy rate (RER) and energy expenditure were significantly increased in HFD + ARB mice than HFD mice (Fig. 3E–H). These effects were more pronounced in a dark environment (Fig. 3A–H). Mice in the HFD + ARB group displayed significantly higher levels of physical activity than HFD mice, and this effect was independent of feed intake (Figs. S3F–G). These results indicated that ARB may improve HFD-induced energy metabolism disorders in mice.

We further investigated the effects of ARB on oxidative stress in mice. We found that, compared with the HFD group, ARB reduced ROS and MDA accumulation, and increased GSH content (Fig. 3I–K), suggesting that ARB could ameliorate HFD-induced oxidative stress. We also examined Fe and Fe^2+^ levels in the liver, and found that HFD caused a significant accumulation in liver Fe^2+^ content without a significant change in Fe content. However, addition of ARB to the diet inhibited the HFD-induced increase in Fe^2+^ levels (Fig. 3L-N), while serum Fe levels showed a similar trend (Fig. 3O).

Finally, qRT-PCR showed that supplementing the diet of HFD mice with ARB led to a significant increase in the expression of genes that inhibit ferroptosis (GPX4, SLC7A11) and decrease in the expression of genes that promote ferroptosis (ACSL4, STA1) compared to HFD mice (Fig. 3P). Western blot analysis revealed that ARB promoted expression of GPX4 and SLC7A11 (Fig. 3Q-R). These findings suggested that ARB ameliorates oxidative stress and alleviates ferroptosis in mice.

### 3.5 FTO is a target of ARB

**Fig. 4:**
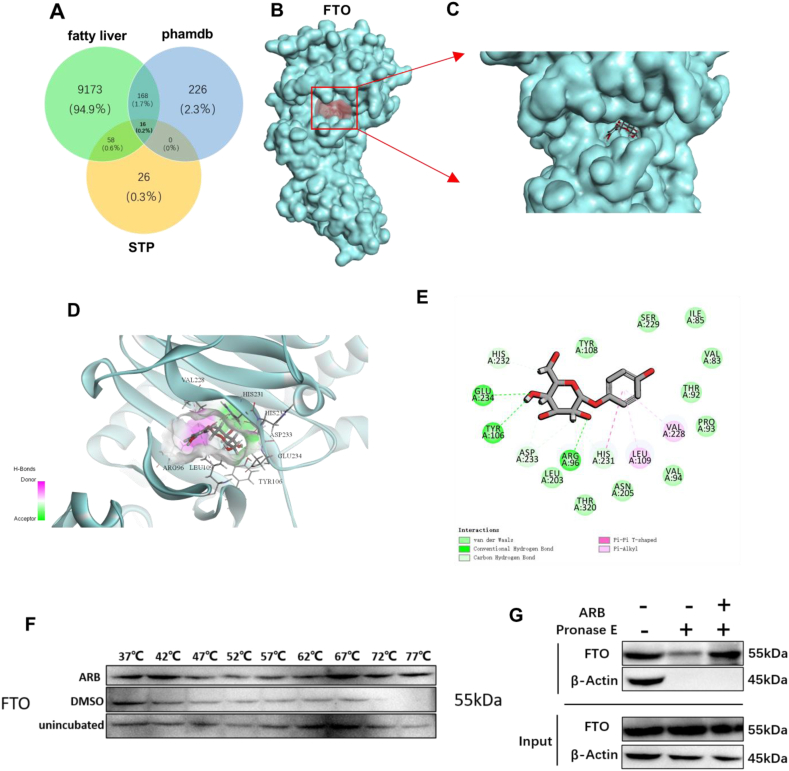
**FTO is a target of ARB.** (A) Venn diagram of screened ARB acting on NAFLD targets. (B–E) Molecular docking results of ARB and FTO. (F) CETSA. (G) DARTS. The FTO is denoted in blue, ARB is denoted in gray and green circles represent hydrogen, light green circles represent hydrophobic force, purple circles represent van der Waals power. (For interpretation of the references to colour in this figure legend, the reader is referred to the Web version of this article.)

Next, we sought to determine the potential mechanisms by which ARB regulates lipid metabolism using Discovery Studio to perform reverse docking analysis. Four hundred and ten potential targets were identified after models with Fitvalues below 0.6 were excluded, and de-duplication and biocorrection were implemented on the prediction data. To refine the target selection, we overlapped targets related to NAFLD obtained from the GeneCards and Swiss TargetPredicion databases with the 410 identified targets, and identified 16 targets that may be potentially associated with the effects of ARB on NAFLD (Fig. 4A). Among the identified targets, the top 10 target proteins included fat mass and obesity-associated protein (FTO), cyclin-dependent kinase 2 (CDK2), and glycogen synthase kinase-3 beta (GSK-3β) (Table S2). We were particularly interested in the demethylating enzyme FTO, since we have previously shown that FTO effectively regulates liver lipid metabolism [20]. Thus, we hypothesized that FTO might be a potential target of ARB that regulates lipid metabolism. To test this, we carried out molecular docking of ARB and FTO, and found that ARB formed direct hydrogen bonding interactions with FTO at Glu234, Tyr106, and Arg96, and hydrophobic interactions with Asp233, His231, and His232 (Fig. 4B–E). Together, these results suggested that FTO is a target of ARB that may mediate its effects on lipid metabolism.

Western blot analysis revealed that ARB had no effect on FTO protein expression levels (Figs. S4A–B), while qRT-PCR analysis demonstrated that ARB inhibited FTO mRNA expression in FTO-overexpressing cells (Fig. S4C). Next, using the cellular thermal shift assay (CETSA) and differentiable architecture search (DARTS), we confirmed that ARB bound to FTO (Fig. 4F and G).

### 3.6 ARB regulates intracellular lipid metabolism and ferroptosis via FTO

**Fig. 5:**
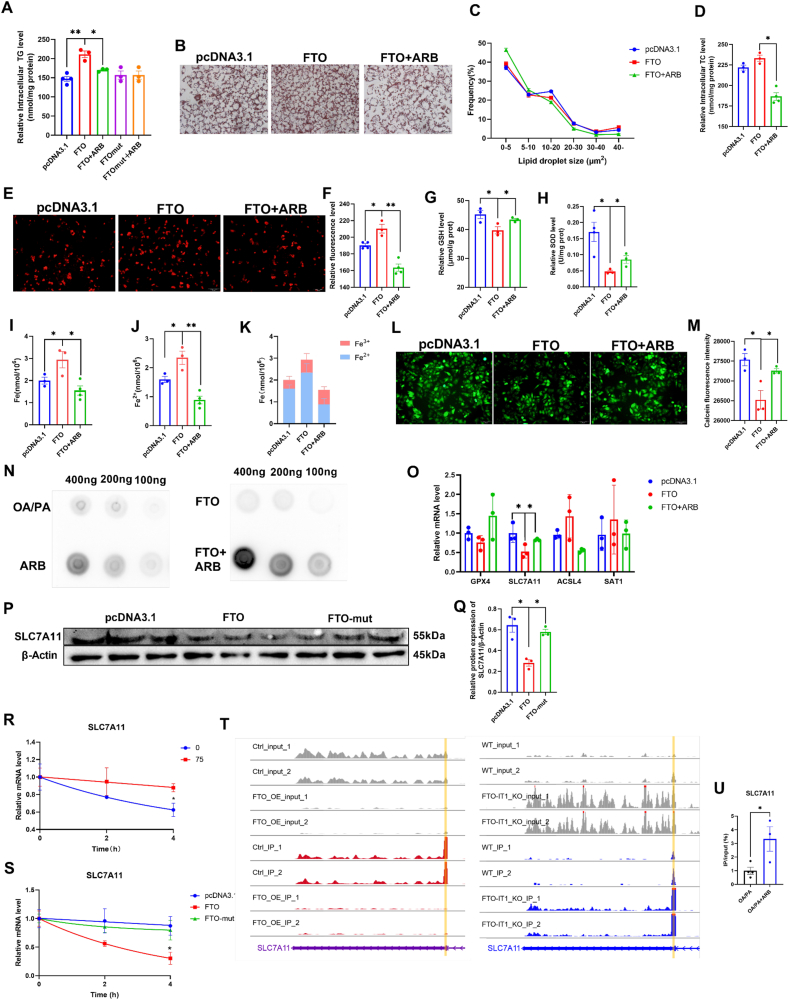
**ARB regulates intracellular lipid metabolism and ferroptosis and promotes methylation modification of SLC7A11 by inhibiting FTO.** (A) The effect of FTO and FTO-mut overexpression on intracellular TG content (n = 3–4 per group). (B–C) Oil red O staining and lipid droplet size analysis. (D) Effects of FTO overexpression on TC content (n = 3–4 per group). (E–F) ROS staining with MitoSOX red probe (n = 3–4 per group). (G–H) Effect of FTO overexpression on intracellular GSH and SOD level (n = 3 per group). (I–K) Intracellular Fe, Fe^2+^ and Fe^2+^ to Fe^3+^ ratio in FTO overexpressing cells (n = 3 per group). (L–M) Calcein staining (n = 3 per group). (N) m6A dot blotting. (O) qPT-PCR of ferroptosis-related genes after FTO overexpression (n = 3 per group). (P–Q) Western Blot detects the effect of FTO overexpression on SLC7A11 protein levels (n = 3 per group). (R–S) Effect of ARB, FTO, and FTO-mut treatments on SLC7A11 mRNA half-life (n = 3 per group). (T–U) Merip-seq as well as Merip-qRCR (X: n = 3–4 per group). Data are mean ± SEM, *n* ≥ 3; One-way ANOVA was used to compare the means of three groups, ∗*P* < 0.05; ∗∗*P* < 0.01. (For interpretation of the references to colour in this figure legend, the reader is referred to the Web version of this article.)

FTO causes disruption of cellular lipid metabolism and energy metabolism, as well as impacts oxidative stress. We found that ARB treatment downregulated the FTO-induced increase in TG content, but had no effect on TG levels in cells overexpressing FTO-mut (loss of demethylation function) (Fig. 5A). Consistent with these findings, Oil red O staining revealed that ARB treatment reduced the abnormal accumulation of intracellular lipid droplets, particularly those with an area larger than 40 μm^2^, induced by FTO overexpression (Fig. 5B and C). Similarly, ARB treatment led to a reduction in the FTO-induced increase in TC levels (Fig. 5D). Together, these results suggested that ARB may affect lipid metabolites through the regulation of FTO demethylase activity.

FTO overexpression increased ROS accumulation, whereas ARB reversed these effects (Fig. 5E–F, Figs. S4D–E). Moreover, ARB treatment of FTO-overexpressing cells increased GSH, SOD, MDA levels and decreased mitochondrial fluorescence (Fig. 5G–H, Figs. S4F–H). The effects of FTO overexpression on cellular Fe and Fe^2+^ content was next evaluated. We found that ARB treatment reversed the FTO-induced increase in Fe^2+^ levels (Fig. 5I–K). Our calcein staining data were consistent with these findings (Fig. 5L-M). Together, these results provided further evidence that the regulatory effects of ARB on lipid metabolism and ferroptosis are mediated through FTO.

### 3.7 ARB promotes m6A methylation of SLC7A11 by inhibiting FTO

Next, we used a dot blotting assay to determine whether ARB affects the FTO-mediated modification of m6A methylation. Higher levels of m6A were observed in the ARB group (Fig. 5N). qRT-PCR analysis demonstrated that ARB increases the FTO-induced reduction in ferroptosis suppressor genes, such as GPX4 and SLC7A11. Conversely, ARB was found to decrease expression of ferroptosis-promoting genes, including ACSL4 and SAT1 (Fig. 5O). Since the decrease in SLC7A11 expression was the most striking, we focused on SLC7A11 in subsequent experiments. Overexpression of FTO led to a reduction in SLC7A11 protein expression levels, while overexpression of FTO-mut had no effect on SLC7A11 expression (Fig. 5P-Q). Next, we examined the half-life of SLC7A11, and found ARB treatment increased the half-life of SLC7A11, thereby promoting its stability (Fig. 5R). FTO overexpression reduced the half-life of SLC7A11, while FTO-mut had no effect (Fig. 5S).

To assess the effects of FTO on the m6A methylation of SLC7A11, we used MeRIP-seq to analyze and identify the methylation sites of SLC7A11. We found that knockdown of FTO led to increased methylation of the 3′-untranslated region (3′-UTR) of SLC7A11, while FTO overexpression resulted in reduced m6A methylation in the 3′-UTR region of SLC7A11 (Fig. 5T). Furthermore, MeRIP-qPCR confirmed that ARB treatment promoted m6A methylation of the 3′-UTR region of SLC7A11 (Fig. 5U). These findings suggested that ARB-mediated inhibition of FTO promoted m6A methylation in the 3′-UTR region of SLC7A11, thereby promoting SLC7A11 expression.

### 3.8 ARB inhibits ferroptosis by affecting the expression of SLC7A11

**Fig. 6:**
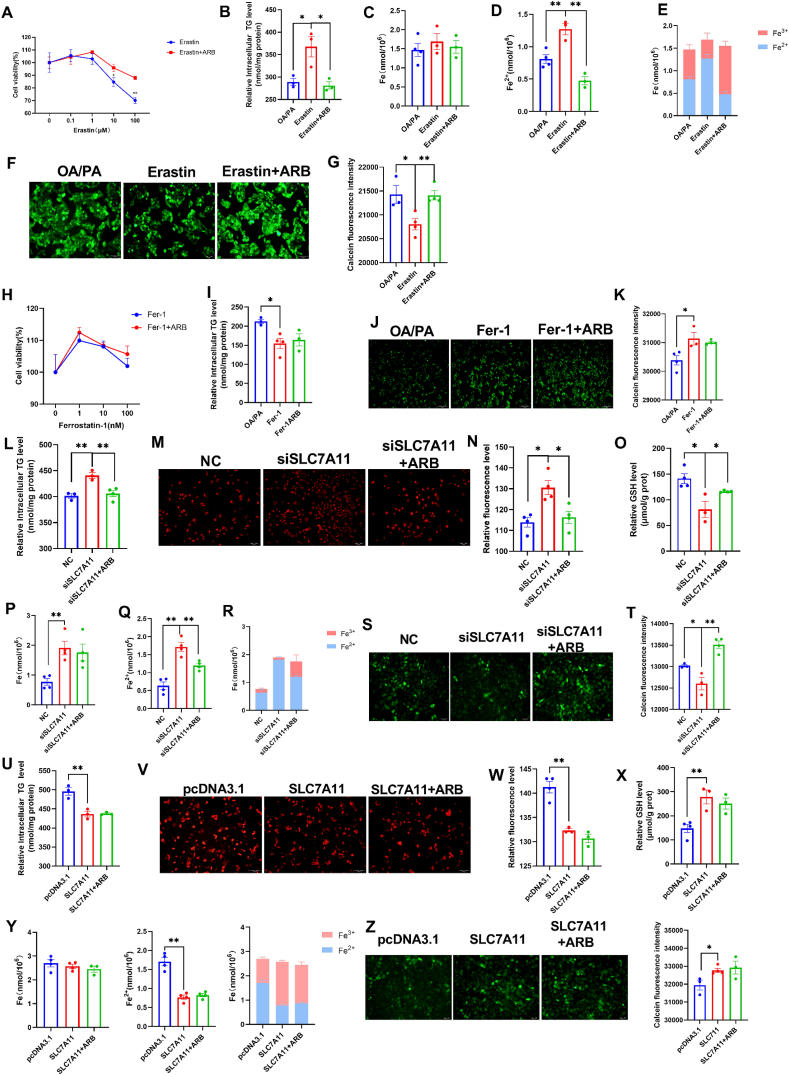
**ARB affects SLC7A11 to inhibit ferroptosis.** (A) CCK8 detects the effect of 0.1–100 μM Erastin treated cells for 24 h on cell viability (n = 3 per group). (B) CCK8 detects the effect of 10 μM Erastin treated cells for 24 h on intracellular TG content (n = 3 per group). (C–E) Fe, Fe^2+^, and Fe^2+^ to Fe^3+^ ratio (n = 3–4 per group). (F–G) Calcein staining (n = 3–4 per group). (H) Effect of 1–100 nM Fer-1 treated cells for 24 h on cell viability (n = 3 per group). (I) Effect of 10 nM Fer-1 treated cells for 24 h on intracellular TG content (n = 3–4 per group). (J–K) Calcein staining (n = 3–4 per group). (L) Effect of siSLC7A11 on intracellular TG content (n = 3–4 per group). (M–O) Effect of siSLC7A11 on intracellular ROS and GSH (n = 3–4 per group). (P–R) Fe, Fe^2+^, and Fe^2+^ to Fe^3+^ ratio (n = 4 per group). (S–T) Calcein staining (n = 3–4 per group). (U) Effect of SLC7A11 overexpression on intracellular TG content (n = 3 per group). (V–X) Effect of SLC7A11 overexpression on intracellular ROS and GSH (n = 3–4 per group). (Y) Fe, Fe^2+^, and Fe^2+^ to Fe^3+^ ratio (n = 4 per group). (Z) Calcein staining (n = 3–4 per group). Data are mean ± SEM, *n* ≥ 3; One-way ANOVA was used to compare the means of three groups, ∗*P* < 0.05; ∗∗*P* < 0.01.

Next, we used Erastin, a specific activator of ferroptosis that interacts with SLC7A11, to examine the role of SLC7A11 in lipid metabolism and ferroptosis. Erastin was found to inhibit SLC7A11 expression by forming a direct hydrogen-bonding interaction with Gln191 (Figs. S5A–C). ARB was found to alleviate 0.1–100 μM Erastin treated cells for 24 h induced cell death as measured by the CCK-8 assay (Fig. 6A). Furthermore, ARB reduced the 10 μM Erastin treated cells for 24 h induced increase in TG (Fig. 6B) and Fe^2+^ (Fig. 6C–E) levels. Our calcein staining data were consistent with these results (Fig. 6F and G). To further validate our findings, we next examined the effects of Ferrostatin-1 (Fer-1), an inhibitor of ferroptosis on cell viability and lipid metabolism. We found that 1–100 nM Fer-1 treated cells for 24 h promoted cell viability, while ARB treatment had no significant effect (Fig. 6H). Similarly, although 10 nM Fer-1 and Fer-1+ARB treatment led to a reduction in extracellular TG levels compared to OA/PA-treated cells, no significant differences between Fer-1 and Fer-1+ARB treatment were observed (Fig. 6I). Our calcein staining data revealed a similar trend (Fig. 6J and K).

We transfected HepG2 cells with SLC7A11 siRNA (siSLC7A11) to knockdown SLC7A11 expression. We found that ARB reversed the siSLC7A11-induced increase in intracellular TG levels (Fig. 6L). In addition, knockdown of SLC7A11 led to ROS accumulation and a reduction in GSH content, both of which were reversed by ARB treatment (Fig. 6M − O, Figs. S6A–B). ARB treatment inhibited the siSLC7A11-induced increase in Fe^2+^ levels (Fig. 6P-R), while our calcein staining data revealed a similar effect (Fig. 6S-T). In contrast, overexpression of SLC7A11 resulted in a decrease in intracellular TG levels (Fig. 6U), together with decreased ROS accumulation and Fe^2+^ levels and increase in GSH levels (Fig. 6V-Z, Figs. S6C–D). No significant differences between the siSLC7A11 and siSLC7A11 + ARB treatment groups were observed, suggesting that ARB acts through SLC7A11.

### 3.9 AAV-mediated SLC7A11 hepatic overexpression alleviates fatty liver in mice

**Fig. 7:**
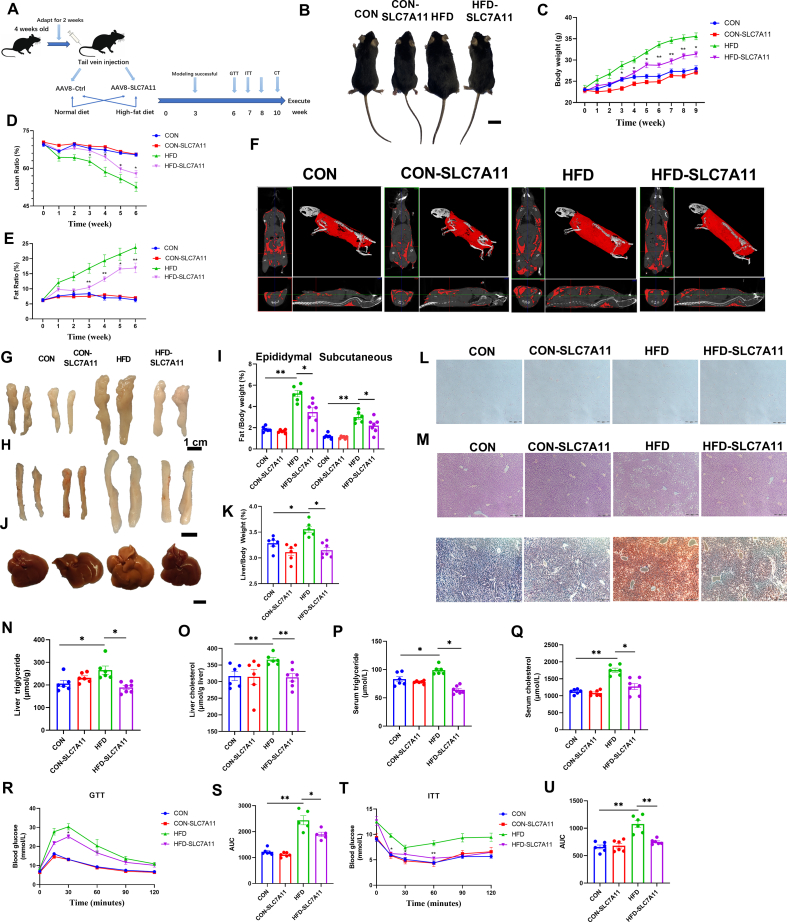
**AAV-mediated SLC7A11 hepatic overexpression alleviates fatty liver in mice.** (A) Schematic diagram of the experimental process in mice. (B) Pictures of mice body size. (C–E) NMR test the body weight and body fat percentage of mice (n = 6–7 per group). (F) CT pictures of mice. (G–I) Pictures of epididymal and subcutaneous fat and relative weights. (J–K) Pictures of liver and relative weights. (L) H&E staining of epididymal fat. (M) H&E staining of liver and oil red O staining. (N–O) Liver TG and TC content in different groups of mice. (P–Q) Serum TG, TC levels in different groups of mice (n = 6–7 per group). (R–S) Mice treated at week 6 for GTT assay (n = 5–6 per group). (T–U) ITT assay at week 7 of mice treatment (n = 6 per group). Data are mean ± SEM, *n* ≥ 6; One-way ANOVA was used to compare the means of four groups, ∗*P* < 0.05; ∗∗*P* < 0.01. (For interpretation of the references to colour in this figure legend, the reader is referred to the Web version of this article.)

To further determine the effects of SLC7A11 on NAFLD, we delivered an AAV vector encoding SLC7A11 into the livers of mice by tail vein injection. Mice were then fed either CON or a HFD. Mice injected with the control AAV were also separated into CON and HFD groups. The experimental process is outlined in Fig. 7A. After 10 weeks, mice were humanely killed and SLC7A11 protein expression levels were measured in liver, heart, spleen, kidney and epididymal fat tissue samples. Our data indicated that SLC7A11 had been successfully expressed in the liver. Western blot analysis further revealed significant increases in SLC7A11 protein expression levels in the epididymal fat and kidneys of HFD-SLC7A11 mice compared to HFD mice (Figs. S7A–K).

We found that the body weight and fat size were significantly lower in the HFD-SLC7A11 group compared to the HFD group, while the lean ratio was significantly higher (Fig. 7B–E). CT scans also revealed that the fat content of the HFD-SLC7A11 group was significantly reduced compared to the HFD mice (Fig. 7F). In addition, the weights of the epididymal fat, subcutaneous fat, and liver were also significantly reduced in the HFD-SLC7A11 group (Fig. 7G–K). Furthermore, overexpression of SLC7A11 in HFD mice resulted in a reduction in fat content and area in epididymal fat tissue, and alleviated tissue damage and lipid deposition in the liver compared to HFD mice (Fig. 7L-M).

A significant decrease in TG and TC levels in the liver and serum was observed in HFD-SLC7A11 mice compared to the HFD group (Fig. 7N-Q). Serum index analysis revealed that LDL-C, AST, and ALT levels were significantly decreased in the HFD-SLC7A11 group compared to the HFD group, while HDL-C levels were significantly increased (Figs. S8A–D). These findings suggested that overexpression of SLC7A11 alleviated NAFLD in mice.

The effects of SLC7A11 on glucose metabolism were examined in mice by performing GTT and ITT tests in the 6th and 7th week of treatment. Our GTT data demonstrated that overexpression of SLC7A11 led to improved glucose tolerance in mice in HFD mice (Fig. 7R and S). Similarly, our ITT data showed that overexpression of SLC7A11 improved insulin resistance in HFD mice (Fig. 7T-U). These findings suggested that SLC7A11 plays a beneficial role in regulating glucose metabolism and improving insulin sensitivity in HFD-fed mice.

### 3.10 AAV-mediated SLC7A11 hepatic overexpression improves energy metabolism and reduces oxidative stress

**Fig. 8:**
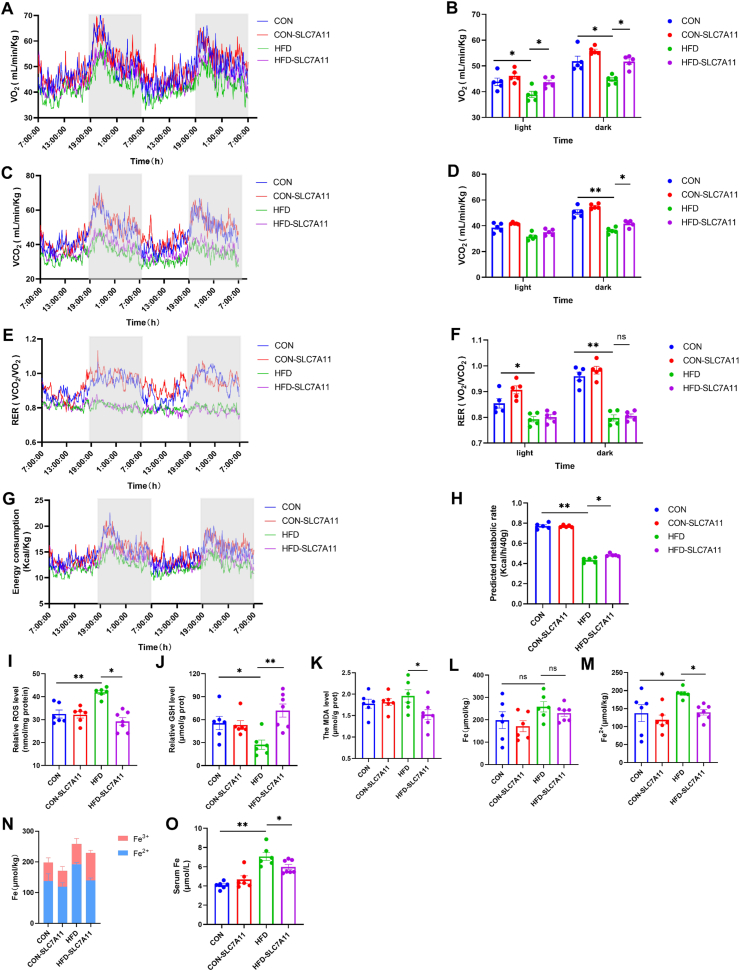
**AAV-mediated SLC7A11 hepatic overexpression improves energy metabolism and reduces oxidative stress.** (A–B) O_2_ consumed (n = 5 per group). (C–D) CO_2_ produced (n = 5 per group). (E–F) RER (VO_2_/VCO_2_) (n = 5 per group). (G–H) Energy metabolism (n = 5 per group). (I–K) Effect of SLC7A11 infection of the liver on ROS, GSH, and MDA (n = 6–7 per group). (L–N) Fe, Fe^2+^, and Fe^2+^ to Fe^3+^ ratio (n = 6–7 per group). (O) Serum Fe levels (n = 6–7 per group). Data are mean ± SEM, *n* ≥ 5; One-way ANOVA was used to compare the means of four groups, ∗*P* < 0.05; ∗∗*P* < 0.01.

The effects of SLC7A11 overexpression on energy metabolism were examined in mice using metabolic cage experiments in the 8th week of treatment. Overexpression of SLC7A11 had no significant effect on energy metabolism in CON mice, but led to a significant improvement in O_2_ consumption and CO_2_ production rate in HFD mice (Fig. 8A–D). Our RER and energy expenditure data were consistent with these findings (Fig. 8E–H). Furthermore, these changes in energy metabolism were more pronounced under dark conditions (Fig. 8A–H), and were independent of feed intake and exercise (Figs. S8E–F).

No significant changes in ROS and GSH levels were observed in the livers of CON mice. However, ROS levels in HFD-SLC7A11 mice were significantly decreased (Fig. 8I) and GSH levels were significantly increased compared to HFD mice (Fig. 8J). Liver MDA levels were also significantly decreased in HFD-SLC7A11 mice compared to HFD mice (Fig. 8K). These findings indicated that overexpression of SLC7A11 could improve energy metabolism and oxidative stress in HFD-fed mice. The Fe^2+^ content in the livers of HFD mice was significantly higher than in CON mice. However, overexpression of SLC7A11 led to a significant reduction in Fe^2+^ levels in HFD-SLC7A11 mice compared to HFD mice (Fig. 8L-N). A similar trend was observed in serum Fe levels (Fig. 8O). These results suggested that overexpression of SLC7A11 in the liver ameliorates ferroptosis.

## 4 Discussion

The two-hit theory was initially proposed in 1998 as a conceptual framework to explain the development of NAFLD [21,22]. According to this theory, the first hit involves the accumulation of fat in the liver and the development of insulin resistance. The second hit involves oxidative stress [23]. This critical link is significant because oxidative stress can result in the peroxidation of cell membrane lipids, mitochondrial dysfunction, and impairment of antioxidant defenses. These processes can further contribute to fat accumulation and liver injury [24]. However, as research on NAFLD has advanced, it has become evident that the classical two-hit theory alone cannot fully elucidate the complex mechanisms underlying NAFLD development and progression. To account for the complexity of NAFLD pathogenesis, a multiple-hit theory has been proposed [25,26]. Novel insights have indicated that epigenetics, iron overload, genetics, mitochondrial dysfunction, intestinal flora, and inflammatory activation are all involved in the progression of NAFLD [[27], [28], [29], [30]]. The dysregulation of epigenetic modifications, particularly m6A RNA methylation, has emerged as a significant contributor to the development of NAFLD [31,32]. Therefore, modulation of m6A methylation provides a new direction for the treatment of NAFLD [33]. In this study, we found that ARB inhibited the demethylase activity of FTO to increase intracellular m6A methylation, thereby reducing fat deposition (Fig. 5A–C, Fig. 5O). These findings may be beneficial for the development of new drugs for the treatment of NAFLD.

Iron metabolism is essential for maintaining the physiological status in humans as well as other animals. Iron homeostasis is primarily maintained by regulating intestinal iron absorption and hepatic iron stores [34]. Iron uptake occurs mainly through SLC11A2-mediated absorption in the small intestine [35] and subsequently enters the circulation, where it is transported by transferrin to various tissues and organs for the synthesis of hemoglobin and iron-dependent enzymes. The exocytosis of iron is mainly accomplished through the intestines and skin, SLC40 is involved in the exocytosis of iron in the intestine [36]. In this regard, Hepatocyte-secreted hepcidin reduces intestinal iron uptake by decreasing the stability of transferrin, and it inhibits the release of stored iron [37].

Iron imbalance has been linked to the development of metabolic diseases. Iron overload has been recognized as a significant factor in the progression of NAFLD [38,39]. Excessive intracellular ferrous ions can lead to liposome peroxidation through the Fenton reaction [40], triggering oxidative stress and alter the metabolism of nutrients in the body, which leads to metabolic disorders and promotes the development of NAFLD [41]. However, supplementation with antioxidants may mitigate the detrimental effects of oxidative stress and disturbed iron metabolism caused by hepatic fat accumulation, thus protecting the liver from damage associated with NAFLD [41,42]. In our study, we observed that ARB treatment was able to enhance the antioxidant effects by increasing SOD and GSH content, while reducing Fe^2+^ accumulation (Fig. 1R–V). As a result, cellular oxidative stress was reduced, leading to a decrease in intracellular fat deposition. These findings suggest that ARB may regulate intracellular iron metabolism, which could potentially attenuate the development of NAFLD.

Recent studies have highlighted the association between epigenetic modifications and iron metabolism in the context of NAFLD [43,44]. The interplay between these two factors can significantly influence the development and progression of NAFLD. m6A RNA methylation, a type of epigenetic modification, has been found to play a role in cellular iron homeostasis by modulating RNA stability and post-transcriptional modifications of genes involved in iron metabolism [45]. m6A modification can serve as a mark to attract binding proteins, which prevents mRNA degradation [46]. However, it should be noted that the impact of m6A modification on mRNA stability is complex and may be regulated by other factors [47]. Recently, METTL3, the major methyltransferase responsible for catalyzing the formation of m6A in mRNA, promotes mRNA decay when recognized by YTH N6-methyladenosine RNA binding protein 2 (YTHDF2) [48]. Consequently, METTL3 was found to accelerate SLC7A11 degradation [49]. However, when it is recognized by insulin-like growth factor 2 mRNA-binding proteins (IGF2BPs), it promotes mRNA stability [50]. FTO is a demethylase that acts in the opposite direction to METTL3. Here, we demonstrated that FTO decreased m6A methylation of the 3′-UTR of SLC7A11, thereby reducing the stability of SLC7A11 (Fig. 5T-U), while ARB, acting as an inhibitor of FTO, promoted SLC7A11 stability, thereby inhibiting ferroptosis.

SLC7A11 (cystine/glutamate antitransporter) is a key subunit in system xc- (cystine/glutamine antiporter system) [51], a key regulatory system of ferroptosis [52]. Here, we demonstrated that SLC7A11 exerts its mitigating effect on NAFLD through a variety of mechanisms, including the regulation of intracellular Fe^2+^ and GSH levels, which lead to a reduction in lipid accumulation (Fig. 6A-Z). Furthermore, overexpression of SLC7A11 *in vivo* ameliorated NAFLD by inhibiting liver ferroptosis (Figs. 7N, Fig. 8L-N). Thus, our data suggested that SLC7A11 mediates GSH synthesis and reduces the susceptibility of liver cells to oxidative stress, as well as ferroptosis, thereby alleviating the development and progression of NAFLD. These findings highlight the important role of SLC7A11 in NAFLD progression and provide novels targets for the development of NAFLD treatment strategies.

In conclusion, the present study reports that ARB, a novel, natural small molecule, ameliorates HFD-induced NAFLD through the FTO/SLC7A11 pathway. Its mechanism of action involves inhibition of FTO, which leads to increased m6A methylation of SLC7A11 and promotes SLC7A11 expression, ultimately inhibiting ferroptosis. To the best of our knowledge, this is the first time that the alleviating effects of SLC7A11 on NAFLD have been reported. Our findings provide a novel approach, as well as a theoretical basis for examining the treatment of NAFLD.

## Author contributions

Lei Zhou and Yixing Li designed the research. Tianyu Jiang, Yao Xiao and Jinfeng Zhou carried out the experiments. Zupeng Luo and Lin Yu conducted data analysis. Qichao Liao, Siqi Liu, Xinyi Qi, Hao Zhang, Menglong Hou, WeiWei Miao, Batbold Batsaikhan, Turtushikh Damba and Yunxiao Liang participated part of the experiments. Tianyu Jiang and Yao Xiao wrote the manuscript. Lei Zhou finalized the manuscript. All of the authors have read and approved the final manuscript.

## Declaration of competing interest

The authors declare that they have no known competing financial interests or personal relationships that could have appeared to influence the work reported in this paper.

## Data Availability

Data will be made available on request.
