# Corrigendum to “Arbutin alleviates fatty liver by inhibiting ferroptosis via FTO/ SLC7A11 pathway” [Redox Biol. 68 (2023) 102963]

**DOI:** 10.1016/j.redox.2023.102974

**Published:** 2023-12-02

**Authors:** Tianyu Jiang, Yang Xiao, Jinfeng Zhou, Zupeng Luo, Lin Yu, Qichao Liao, Siqi Liu, Xinyi Qi, Hao Zhang, Menglong Hou, WeiWei Miao, Batbold Batsaikhan, Turtushikh Damba, Yunxiao Liang, Yixing Li, Lei Zhou

**Affiliations:** a Institute of Digestive Disease, Guangxi Academy of Medical Sciences, The People's Hospital of Guangxi Zhuang Autonomous Region, Nanning, 530021, China; b College of Animal Science and Technology, Guangxi University, Nanning, 530004, China; c Department of Internal Medicine, Institute of Medical Sciences, Mongolian National University of Medical Sciences, Ulaanbaatar, Mongolia; d Department of Health Research, Graduate School, Mongolian National University of Medical Sciences, Ulaanbaatar, Mongolia; e School of Pharmacy, Mongolian National University of Medical Sciences, Ulaanbaatar, Mongolia

The authors regret < The spelling error of the co-first author “Yao Xiao”. The correct name should be “Yang Xiao”>.

The authors would like to apologise for any inconvenience caused.

