# Supplementary material for: Arbutin alleviates fatty liver by inhibiting ferroptosis via FTO/SLC7A11 pathway
Source: Redox Biol. 2023 Nov 16;68:102963. doi: 10.1016/j.redox.2023.102963 (PMC10694775; doi:10.1016/j.redox.2023.102963)
Supplement: Multimedia component 1 [file mmc1.docx]

**Supplementary Material for**

**Original article**

**Arbutin alleviates fatty liver by inhibiting ferroptosis via FTO/SLC7A11 pathway**

**Authors:** Tianyu Jiang^1†^, Yao Xiao^1†^, Jinfeng Zhou^2^, Zupeng Luo^1^, Lin Yu^1^, Shuning Lai^1^, Xinyi Qi^1^, Hao Zhang^1^, Menglong Hou^1^, WeiWei Miao^1^, Batbold Batsaikhan^3,4^, Turtushikh Damba^5^, Yunxiao Liang^1^, Yixing Li^2^*, Lei Zhou^1^*

**Author affiliations:** 1. Institute of Digestive Disease, Guangxi Academy of Medical Sciences, the People's Hospital of Guangxi Zhuang Autonomous Region, Nanning 530021, China. 2. College of Animal Science and Technology, Guangxi University, Nanning 530004, China. 3. Department of Internal Medicine, Institute of Medical Sciences, Mongolian National University of Medical Sciences, Ulaanbaatar, Mongolia. 4. Department of Health Research, Graduate School, Mongolian National University of Medical Sciences, Ulaanbaatar, Mongolia. 5. School of Pharmacy, Mongolian National University of Medical Sciences, Ulan Bator, Mongolia.

***Corresponding author:** Prof. Lei Zhou, E-mail: zhoulei@gxu.edu.cn, Institute of Digestive Disease, Guangxi Academy of Medical Sciences, the People's Hospital of Guangxi Zhuang Autonomous Region, Nanning 530021, China; Prof. Yixing Li, E-mail: liyixing39@gxu.edu.cn, College of Animal Science and Technology, Guangxi University, Nanning 530004, China.

^†^ Tianyu Jiang and Yang Xiao contributed equally to this work.

**1.Materials and Methods**

1.1 Animals and Experimental Design

The animal experiments in this study complied with animal care laws and guidelines. All procedures were approved by the Guangxi University Laboratory Animal Ethics Committee (GXU-2023-0094). A total of 43 male San C57BL/6 mice (SPF Bio-Tech Co., Ltd., Beijing, China), aged six weeks, were obtained from a commercial hatchery. The mice were housed under standard conditions, with a temperature of 25±2°C and a relative humidity of 50±5%. A 12-hour on/off lighting cycle was implemented using incandescent lamps. The mice were provided with ad libitum access to feed and water throughout the study.

To investigate the effects of arbutin on high-fat diet-induced obesity, randomly assigned mice were divided into three groups (n= 6 per group): (1) Control group, fed a basic diet; (2) HFD group, fed a high-fat diet; (3) HFD+ARB group, feeding add 1g ARB per kg of high- fat diet.

The part of the mouses were randomly divided into four groups (n= 6-7 per group). (1): Control group: tail vein injection of control viral fluid and feed basic diet; (2): Control-AAV8 group: tail vein injection of SLC7A11 viral fluid and feed basic diet; (3): HFD group: tail vein injection of control viral fluid and feed high-fat diet; (4): HFD-AAV8 group: tail vein injection of SLC7A11 viral fluid and feed high-fat diet. The basic diet (TP2330055MC, 3.8 kcal/g, 10% fat calories, 14% protein calories, 76% carbohydrate calories), The high-fat diet (TP2330055M, Calories: 5.5 kcal/g, 60% fat calories, 14% protein calories, 26% carbohydrate calories) and ARB customized feeds were sourced from Trophic Bio-Tech Co., Ltd. Nantong, China.

1.2 Nuclear magnetic resonance (NMR)

Using nuclear magnetic resonance (NMR) technology, (Niumag QMR23-060H-I instrument Suzhou, China), the fat and lean meat rates of mice were measured over eight weeks. Prior to each measurement, the machine was calibrated and the body weight of the mice was entered into the system. During the test, the mice were placed into the instrument and the test was initiated. This testing process was repeated once a week for a period of eight weeks.

1.3 Glucose tolerance test

At week 9 of the experiment, 6 mice were randomly selected from each group and fasted for 16 hours. A tail vein blood sample was collected to test for fasting blood glucose levels in mice. Next, each mouse was injected with 2 g/kg of glucose, and blood glucose levels were measured at 15, 30, 60, 90, and 120 minutes after injection.

1.4 Insulin tolerance test

At week 9 of the experiment, 6 mice were randomly selected from each group and fasted for 4 hours. A tail vein blood sample was collected to test for fasting blood glucose levels in mice. Next, each mouse was injected with 0.6 U/kg of insulin, and blood glucose levels were measured at 15, 30, 60, 90, and 120 minutes after injection.

1.5 Metabolic cage

At least 3 mice are randomly selected from each group for metabolic cage (Promethion Cages, Sable Systems International, Las Vegas, Nevada, U.S.A.) experiments. Metabolic cages recorded exercise, O_2_ consumption, CO_2_ production, respiratory exchange rate (RER), and energy expenditure at 5 minutes intervals for 48 hours as previously described.

1.6 Micro-CT

After anesthetizing mice with tribromoethanol, scanned the mice using Micro-CT (SkyScan 1278, Bruker, Billerica, Massachusetts, U.S.A.) to detect the body fat percentage.

1.7 Cells and cells cultures

HepG2 cells were cultured in Dulbecco's modified Eagle medium (DMEM) supplemented with 10% fetal bovine serum (FBS) and 1% antibiotics (penicillin-streptomycin liquid) at 37 °C in a 5% CO_2_ cell culture incubator. Arbutin (purity >99.96%) was purchased from Yuanye Bio-Technology Co., Ltd. (Shanghai, China).

To culture HepG2 cells, DMEM supplemented with 10% FBS was used to achieve 80% confluency, followed by a 6-hour resting period in serum-free DMEM. Cells were then treated with OA/PA DMEM (OA:200μM PA:100μM) containing ARB (0-100μM) for 24 hours.

1.8 Plasmid constructs and transfection

The full-length FTO and SLC7A11 fragment was loaded from cDNA onto the pcDNA 3.1 vector using In-Fusion Cloning method and produced by transforming DH5α (Sangon Biotech Co., Ltd. Shanghai, China) competent cells into Escherichia coli. After overnight incubation at 37°C, monoclonal colonies were picked out for colony PCR and verification was performed using agarose gel electrophoresis. The constructed plasmids were sequenced.

The pcDNA 3.1 and FTO or SLC7A11 plasmids were transfected into cells using liposomes and incubated with DMEM medium for 6 hours, followed by conversion to OA/PA medium or OA/PA medium containing 75 μM ARB. Lysis detection was performed after 24 hours.

1.9 Sample preparation

At the 12 weeks of experiment, mice were anesthetized and their eyeballs were collected and removed. The hearts, livers, spleen, lungs, kidneys, intestines, and muscle tissues of the mice were also collected. Small steel balls and lysate were added for crushing, followed by overnight shaking at 4°C. The mixture was then centrifuged at 12,000g and 4°C to take the supernatant for testing. Cells were lysed using RIPA (Solarbio Science & Technology Co., Ltd. Beijing, China) containing 1% PMSF (Solarbio Science & Technology Co., Ltd. Beijing, China) or supplemented with PBS (Phosphate-Buffered Saline) ultrasonic disruption, supernatant taken after centrifugation for testing.

1.10 Cell proliferation and cytotoxicity assay

After the cell seeding and 96-well plate growth to 80% confluency, treat with 0, 10, 25, 50, 75, 100 μM arbutin for 24h. Using the CCK-8 assay kit (Yeasen Biotechnology Co., Ltd. Shanghai, Chain) cell viability is detected after 1 h of co-incubation with cells.

1.11 Cell scratch test

Cells were seeded in 6-well plates, divided into control groups and treated groups with 75 μM ARB (Yuanye Bio-Technology Co., Ltd. Shanghai, China). When cells reached confluence, a scratch was made across the cell monolayer using a pipette tip. Observe and acquire images using a microscope at 0, 24, 48, 62 h, respectively.

1.12 Tissue/cell TG, TC assay

Tissue samples from mice were lysed with anhydrous ethanol and crush using a tissue crusher. Subsequently tested according to the manufacturer's method and corrected for the weight of the liver. Cell samples were centrifuged with RIPA lysate containing 1% PMSF at 4°C, subjected to lysis for 30 minutes. Subsequently tested according to the manufacturer's method and detected protein concentrations for normalization. TG and TC values of the cells and tissue samples were assessed by the TG and TC kits (Jiancheng Co., Ltd. Nanjing, China).

1.13 Serum assay (HDL-C, LDL-C, AST, ALT, Fe)

After blood collection from mice, the blood was allowed to set aside at 4°C overnight, and the serum was centrifuged at 3000g. The HDL-C, LDL-C, AST, ALT, and Fe test kits (Jiancheng Co., Ltd. Nanjing, China) were used according to the manufacturer's instructions.1.14 Oil red O staining

After cell culture, the cells were fixed with 4% paraformaldehyde. Afterwards, the fixed cells were treated with a working solution (oil red O stock solution diluted in dd water at a ratio of 3:2) for 30 minutes. The cells were then quickly washed with 60% isopropanol and then washed three times with PBS, taking care not to discard the PBS during the final wash.Photographs were taken using an inverted microscope (IX53; Olympus Corporation, Tokyo, Japan), and quantification was performed by measuring the amount of dye present after the isopropanol washes.

1.15 Glucose metabolism assay (Glycogen, Glucose uptake, Glucose output)

Cells were seeded into 12-well plates, and the glycogen content in the cells was measured using the anthrone method. To extract the glycogen, the cells were collected and sonicated before being boiled in a water bath for 20 minutes. The resulting mixture was centrifuged at 8,000g for 10 minutes at 25°C, and the supernatant was collected for subsequent testing.

The glycogen content was measured using a glycogen content test kit from Solarbio (Solarbio Science & Technology Co., Ltd. Beijing, China). The assay solution was prepared according to the manufacturer’s instructions.

Cells were seeded into 24-well plates and processed for 24 hours. Afterward, they were treated with sugar-free OA/PA containing 2-NBDG (10 μM) and incubated for 2 hours. The absorbance values at the excitation and emission wavelengths were measured using a microplate reader.

The cells were inoculated in 24-well plates and treated with OA/PA or OA/PA medium containing 75 μM ARB for 24 h. The medium was replaced with MEM medium containing 20 μM sodium lactate and 2 μM sodium pyruvate for 4 h. The glucose content of the medium was measured using the Nanjing Glucose Content Assay Kit.

Upon completion of the assay, the protein concentration was determined utilizing the BCA method for homogenization.

1.16 Enzyme assays and biochemical reagents (ROS MDA GSH SOD)

Reactive oxygen species (ROS) and Superoxide dismutase (SOD) kits were purchased from Beyotime Biotechnology, while the glutathione (GSH) kit was obtained from Nanjing Jian Cheng Institute of Bioengineering. The MDA ELISA Kit (Elabscience Biotechnology Co., Ltd, Wuhan, China) used to detect of intracellular Malondialdehyde (MDA) level. After collecting tissue or cells, the assay was performed according to the manufacturer’s instructions. Each experiment was repeated at least three times, and values were normalized against the protein concentration.

1.17 Mitochondrial ROS test

Cellular mitochondrial ROS content was assayed using MitoSOX (Thermo Fisher, Waltham, MA). After cell treatment, 5 μM MitoSOX was used to co-incubate with the cells for 10 min at 37°C, and the cells were washed twice using PBS before observing the cell fluorescence intensity under an inverted microscope (IX53; Olympus Corporation, Tokyo, Japan) and fluorescence quantification was measured using a microplate reader, and the proteins were detected using the BCA method for normalization.

1.18 Tissue ROS and MDA assay

The ROS and MDA levels in the liver were assessed using Mice ROS ELISA KIT (Enzyme-linked Biotechnology Co., Ltd. Shanghai, China) and MDA ELISA Kit (Elabscience Biotechnology Co., Ltd, Wuhan, China). Liver tissue was homogenized using PBS, centrifuged and the supernatant was taken and assayed according to the manufacturer's instructions, and the proteins were detected using the BCA method for normalization.

1.19 Mitochondrial staining

Cells were seeded onto a 24-well plate and then the Mito-Tracker Green (Beyotime Biotechnology, Beijing, China) was diluted in a ratio of 1:10,000 using PBS as a working solution. The working solution was then incubated with the cells for 30 minutes. Following this, the cells were washed three times with PBS, taking care not to discard the PBS during the final wash. Photographs were taken using an inverted microscope (IX53; Olympus Corporation, Tokyo, Japan), and fluorescence quantification was measured using a microplate reader. Protein concentration was determined using homogenization.

1.20 ATP assay

Cells were seeded onto a 12-well plate, and the ATP content was measured using an ATP assay kit (Beyotime Biotechnology Beijing, China). The ATP lysate and ATP detection working solution were prepared in accordance with the manufacturer's instructions. The cells were lysed using the ATP lysate, followed by centrifugation at 4°C and 12,000g for 5 minutes. Working solution was then added to the detection well to consume the original ATP at the bottom before adding 20 μl of the sample per well. The samples were then measured using a luminometer.

Finally, the protein concentration was determined using homogenization.

1.21 Mitochondrial membrane potential assay

The enhanced mitochondrial membrane potential assay kit with JC-1 was purchased from Beyotime Biotechnology. The staining working solution was prepared following the manufacturer's instructions and then co-incubated with the cells at 37°C for 20 minutes. After washing with the staining buffer, the cells were observed using a fluorescence microscope, and the levels of JC-1 monomer and polymer were quantified.

1.22 Tissue and cell iron ion/ferrous ion measure

According to the manufacturer's instructions, tissue and cell iron/ferrous ion concentrations used iron ion assay kits and ferrous ion assay kits (Elabscience Biotechnology Co., Ltd, Wuhan, China) Results were normalized to the number of cells.

1.23 Calcein staining

The Calcein staining kit was purchased from Beyotime Biotechnology. The staining working solution was prepared according to the manufacturer's instructions and co-incubated with the cells at 37°C for 30 minutes. After that, the growth medium was replaced and the cells were incubated at 37°C for an additional 30 minutes. The fluorescence intensity was then detected based on the excitation and emission light.

1.24 Quantitative Real-time PCR

Total RNA was extracted with trizol (GenStar, Beijing, China.) reagent and reverse transcribed into cDNA. After reverse transcription, Real-time quantitative reverse transcription PCR (qRT-PCR) was subsequently performed, using the following reaction system: 5 μL of cDNA, 10 μL of Realstar Green Fast mixture (GenStar, Beijing, China), 1 μL each of a single upstream and downstream primer, and 3 μL of double-distilled water. The primer sequences used in this study are shown in the table S1.

1.25 Molecular docking

The ARB structure was searched through NCBI and imported into Discovery Studio to generate the 3D structure. Preprocessing was performed to correct bond angles and bond orders, followed by CHARMM force field minimization using Discovery Studio. Subsequent reverse target finding using the PharmaDB database generated potential targets for ARB. Protein structures were downloaded through the RCSB protein structure database (https://www.rcsb.org/). All water molecules and heteroatoms were removed and the protein preparation protocol was then optimized in Discovery Studio. Molecular docking was performed using the CDOCKER program in Discovery Studio to assess the binding affinity of the protein to the ligand. The program is based on the molecular docking method and runs within CHARMM. The program docks ligands to protein binding sites and uses high temperature molecular dynamics to generate random ligand conformations. Ten conformations are generated for each ligand and the optimal docking pose is selected based on the highest score and key residue non-bonding interactions.

1.26 Western-blot

To extract the total protein, a lysis buffer containing 1% protease inhibitor was employed, followed by detection using the BCA protein detection kit. After detection, the sample was heated at 100℃ for 10 minutes with 4× protein loading buffer, and then subjected to Sodium salt (SDS)-polyacrylamide gel electrophoresis for separation. The separated protein samples were then transferred onto a polyvinylidene fluoride (PVDF) membrane and blocked by TBST containing 5% skimmed milk for 1 hour. Subsequently, primary antibodies (FTO purchased from Santa Cruz Biotechnology, m6A purchased from Proteintech Group; SLC7A11, GPX4 purchased from Abways Technology Co., Ltd) were added and incubated overnight at 4℃, following which the PVDF membrane was treated five times with TBST. Secondary antibodies (Service biotechnology Co., Ltd, Wuhan, China.) were then incubated for 1 hour and subsequently washed five times with TBST. Finally, ECL hypersensitive luminescent solution (Solarbio Science & Technology Co., Ltd. Beijing, China) was used to detect protein expression, which was further quantitatively analyzed using Image Lab.

1.27 m6A dot blot assay

After total RNA extraction, the samples were diluted to 400, 200, and 100 ng. mRNA samples were denatured at 95°C for 3 minutes, and then incubated on ice for 5 minutes. The samples were then loaded onto a nitrocellulose filter membrane (Solarbio Science & Technology Co., Ltd. Beijing, China) along with 20×SSC buffer (Beyotime Biotechnology, Beijing, Cnina). The membrane was crosslinked under UV light for 5 minutes and washed with PBST three times. After blocking with 5% non-fat milk, the membrane was incubated with a specific m6A antibody at 4°C overnight. The membrane was then incubated with HRP-conjugated anti-mouse IgG for 1 hour and observed using the BIO-RAD Gel Doc XR system.

1.28 Cellular thermal shift assay (CETSA)

Cells were extracted using M-PER (Solarbio Science & Technology Co., Ltd. Beijing, China), and the cell supernatant was divided into three groups after centrifugation. Two groups were incubated with the same concentration of DMSO (Solarbio Science & Technology Co., Ltd. Beijing, China) and ARB at 37°C for 2 hours, and then divided into PCR tubes. The protein was denatured using a PCR instrument at the corresponding temperature for 3 minutes. After centrifuging at 12,000 g at 4°C for 20 minutes, the supernatant was mixed with loading buffer in a ratio of 3:1 and boiled at 100°C. The obtained samples were subjected to Western Blot.

1.29 Differentiable Architecture Search (DARTS)

Untreated HepG2 was lysed using M-PER on ice for 10 min at 4°C and centrifuged at 12,000g for 10 min. The protein final concentration was diluted to 5 μg/μl using 1× TNC (50 mmol/L Tris, 50 mmol/L NaCl, 10 mmol/L CaCl2, pH = 7.4) solution. equal amounts of DMSO and 75 μM ARB were added to the lysed samples and incubated at 37°C for 1 h. The samples were then incubated for 30 min using a ratio of streptavidin to total protein of 1:1750. 4× loading buffer was added to the lysed samples and boiled for 10 min, and the resulting samples were subjected to Western Blot.

1.30 mRNA half-life assay

Cells were seeded in 24-well plates and treated with ARB for 24 hours. Actinomycin D (Aladdin Bio-Technology Co. Ltd. Shanghai, China) was then added to the cells, which served as a transcription inhibitor. The cells were collected at 0 hours (immediately after Actinomycin D addition), 2 hours, and 4 hours after treatment. RNA extraction was performed on the collected cells, and the RNA was reverse transcribed into cDNA. PCR was then performed using fluorescent probes. Using least-square analysis, the best-fit lines were identified for each time point, and the RNA half-life was calculated.

1.31 MeRIP-seq and MeRIP-qPCR

MeRIP-seq results from cells overexpressing or suppressing FTO from others study were used to analyze the m6A methylation site of SLC7A11 (GEO accession: GSE154561, GSE189465). The MeRIP procedure was performed according to the instructions provided by the manufacturer using a MeRIP™ m6A Transcriptome Profiling Kit (RIBOBIO ribo, Guangzhou, China). First, total RNA was extracted from the cells and fragmented by incubation with a 94°C PCR machine for 3 minutes. The fragmented RNA was then precipitated overnight with sodium acetate and glycogen at -20°C, and the resulting precipitate was collected by ethanol extraction. Anti m6A magnetic beads were prepared from Magnetic beads A/G and m6A antibodies, and the RNA was immunoprecipitated with the supernatant of the precipitation products using these beads. After full washing, the RNA was recovered, and subsequent q-PCR detection was performed to analyze the m6A methylation site of SLC7A11.

1.32 Statistical Analysis

The data between two groups were analyzed by independent samples t-test, and one-way ANOVA was used between three or more groups. Data were presented as means ± SEMs from three independent experiments. The differences were considered statistically significant if (∗) *p*< 0.05 or (∗∗) *p*< 0.01.

**2. Supporting tables**

**Table S1 Summary of primer sequences**

| Primers | Forward (5′–3′) | Reverse (5′–3′) |
| --- | --- | --- |
| CD36 | CTTTGGCTTAATGAGACTGGGAC | GCAACAACACACACACACACACAC |
| ACC1 | TTCACTCCACCTTGTCAGCGGA | GTCAGAGAAGCAGCCCATCACT |
| FASN | CGCGTGGCCGGCTACTCCTAC | CGGCTGCCACACACCTCCTCT |
| CPT1 | TGGCATCATCACTGGTGTGTT | GTCTAGGGTCCGATTGATCTTTG |
| PPARα | TGAACAAAGACGGGATG | TCAAACTTGGGTTCCATGAT |
| SCD1 | GCCCCTCTACTTGGAAGACGA | AAGTGATCCCATACAGGGCTC |
| MTTP | GCTAAGAAGCTGATAATGGGAGG | CCACTCTTGGAGAAACGGTCATA |
| APOB | GCTGGGTGCAGACGCTTT | TGCCGTCAGTTCTTGTGACT |
| 28S rDNA | AGGACCCGAAAGATGGTGAACTA | CGGAGGGAACCAGCTACTAGAT |
| ComplexII | CAAACCTACGCCAAAATCCA | GAAATGAATGAGCCTACAGA |
| β-actin | CGACAGGATGCAGAAGGAGAT | CAAGAAAGGGTGTAACGCAACTA |
| GPX4 | TTCCCGTGTAACCAGTTCG | CGGCGAACTCTTTGATCTCT |
| FSP1 | CCTGGGAAAAAGGACAGATGAA | CATGGCAATGCAGGACAGGA |
| SLC7A11 | TGCTGGGCTGATTTTTCTCCG | GAAAGGGCAACCATGAAGAGG |
| HO-1 | GTGCCACCAAGTTCAAGCAG | CACGCATGGCTCAAAAACCA |
| ACSL4 | CATCCCTGGAGCAGATACTCT | TCACTTAGGATTTCCCTGGTCC |
| ALOX5 | CCTCAGGCTTCCCCAAGT | GAAGATCACCACGGTCAGGT |
| SAT1 | CCGTGGATTGGCAAGTTATT | TCCACCCTTCACTGGA |
| NOX2 | TGGAGTTGTCATCACGCT GTG | CTGCCCACGTACAATTCGTTC |
| TF | GTGTGCAGTGTCGGAGCAT | CATCGGATGGAATGACGCTTT |
| TFRC | ACCATTGTCATATACCCGGTTCA | CAATAGCCCAAGTAGCCAATCAT |
| Hepcidin | TGACCAGTGGCTCTCTTTTCC | CGCAGCAGAAAATGCAGATG |
| Fpn-1 | GCCTACTTGTGCCTCCCAGAT | TACCCTGTGGTGATGCAGTCA |
| *Slc7a11* | GCTCGTAATACGCCCTGGAG | GGAAAATCTGGATCCGGGCA |
| *Gpx4* | GCCTGGATAAGTACAGGGGTT | CATGCAGATCGACTAGCTGAG |
| *Acsl4* | CCGACCTAAGGGAGTGATGA | CCTGCAGCCATAGGTAAAGC |
| *Sat1* | TCTTGCCACTTCTTAGCTGC | TTTAGTTACCTCCCCGCCCA |
| *β-actin* | AAGAGCTATGAGCTGCCTGA | TACGGATGTCAACGTCACAC |

**Table S2 Reverse docking screening Top 10 ranking list of target proteins**

| PDB ID | Protein | Fit value |
| --- | --- | --- |
| 6GUE | Cell division protein kinase 2 (CDK2) | 0.908491 |
| 4CXW | Fat mass and obesity-associated protein (FTO) | 0.866006 |
| 1H8F | Glycogen synthase kinase-3 beta (GSK3β) | 0.845374 |
| 3OHL | Matrix metalloproteinase-3(MMP-3) | 0.840198 |
| 8EMS | Glycogen phosphorylase (PYGL) | 0.831534 |
| 1A85 | Matrix metalloproteinase-8 (MMP-8) | 0.826229 |
| 1GKC | Matrix metalloproteinase-9 (MMP-9) | 0.77219 |
| 1A4L | Adenosine aminohydrolase (ADA) | 0.769498 |
| 1WXS | Serine/threonine-protein kinase pim-1 (PIM-1) | 0.766503 |
| 4PL3 | Endoplasmic reticulum-to-nucleus signaling 1 (ERN1) | 0.761173 |

**3. Supporting figures**

**
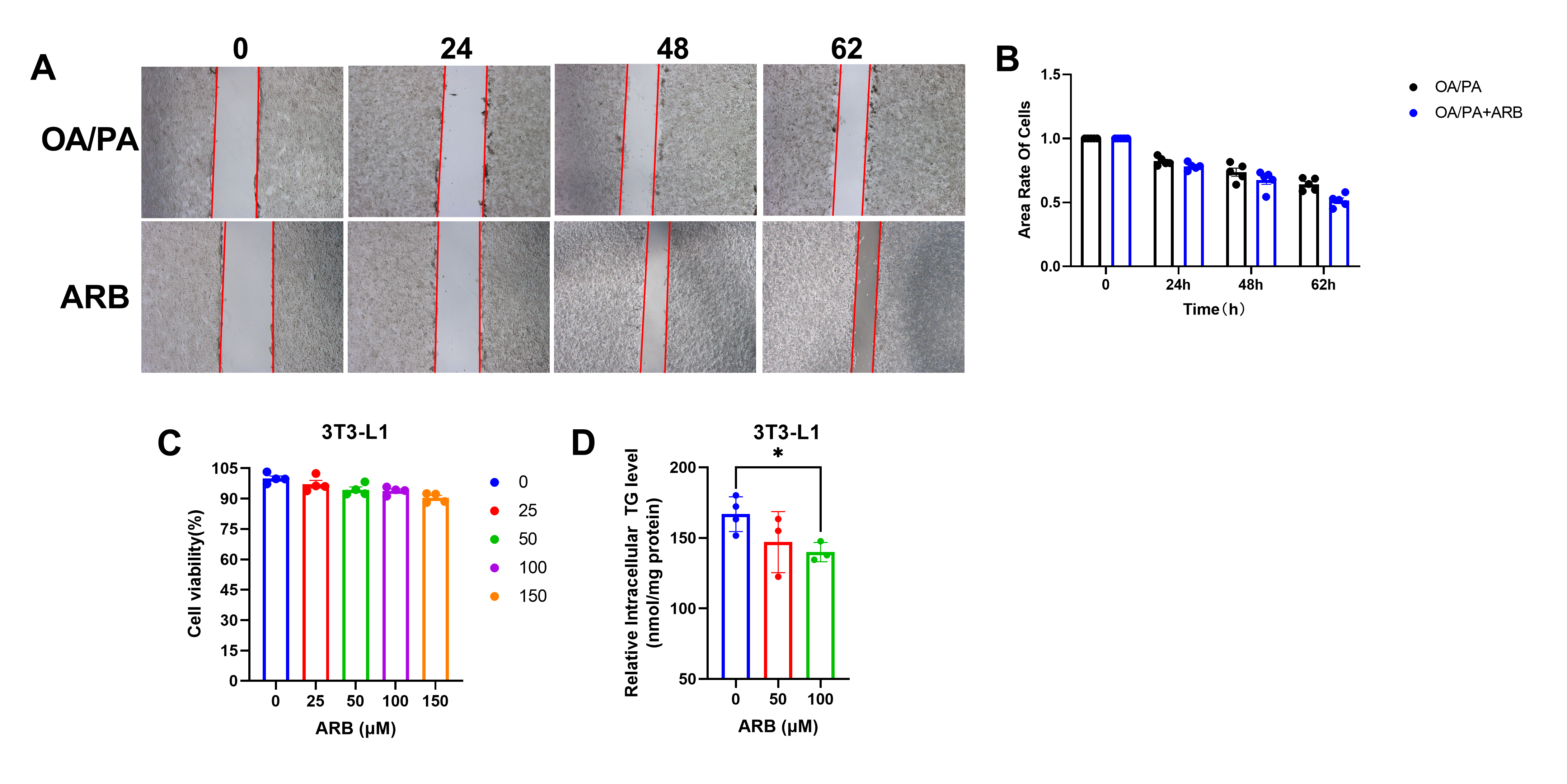
**

**Figure S1. Effect of ARB on cell viability as well as fat deposition.** (A-B) Cell scratch assay and its quantification. (C) Effect of different concentrations of ARB on cell viability of 3T3-L1 cells (n=4 per group). (D) Effects of different concentrations of ARB on intracellular TG in 3T3-L1 cells (n=3-4 per group). Data are mean ± SEM, *n* ≥ 3; The data between two groups were analyzed by independent samples t-test, and one-way ANOVA was used between three or more groups, ∗*P* < 0.05; ∗∗*P* < 0.01

**
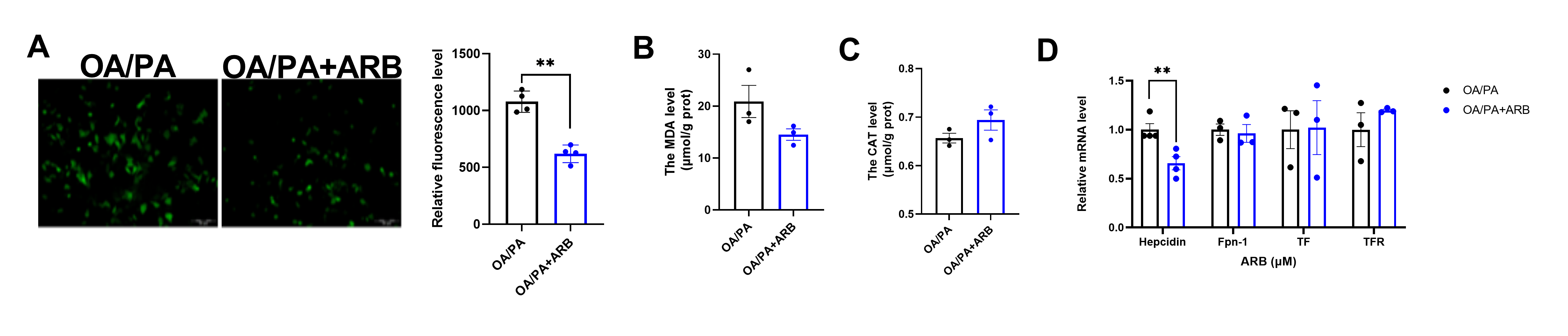
**

**Figure S2. Effect of ARB on ferroptosis.** (A) ROS staining with the DCFH-DA probe (n=4 per group) (B) Effect of ARB on intracellular MDA (n=3 per group). (C) Effect of ARB on intracellular CAT (n=3 per group). (D) Transferrin-related gene qRT-PCR (n=3-4 per group). Data are mean ± SEM, *n* ≥ 3; The data were analyzed by independent samples t-test, ∗*P* < 0.05; ∗∗*P* < 0.01.


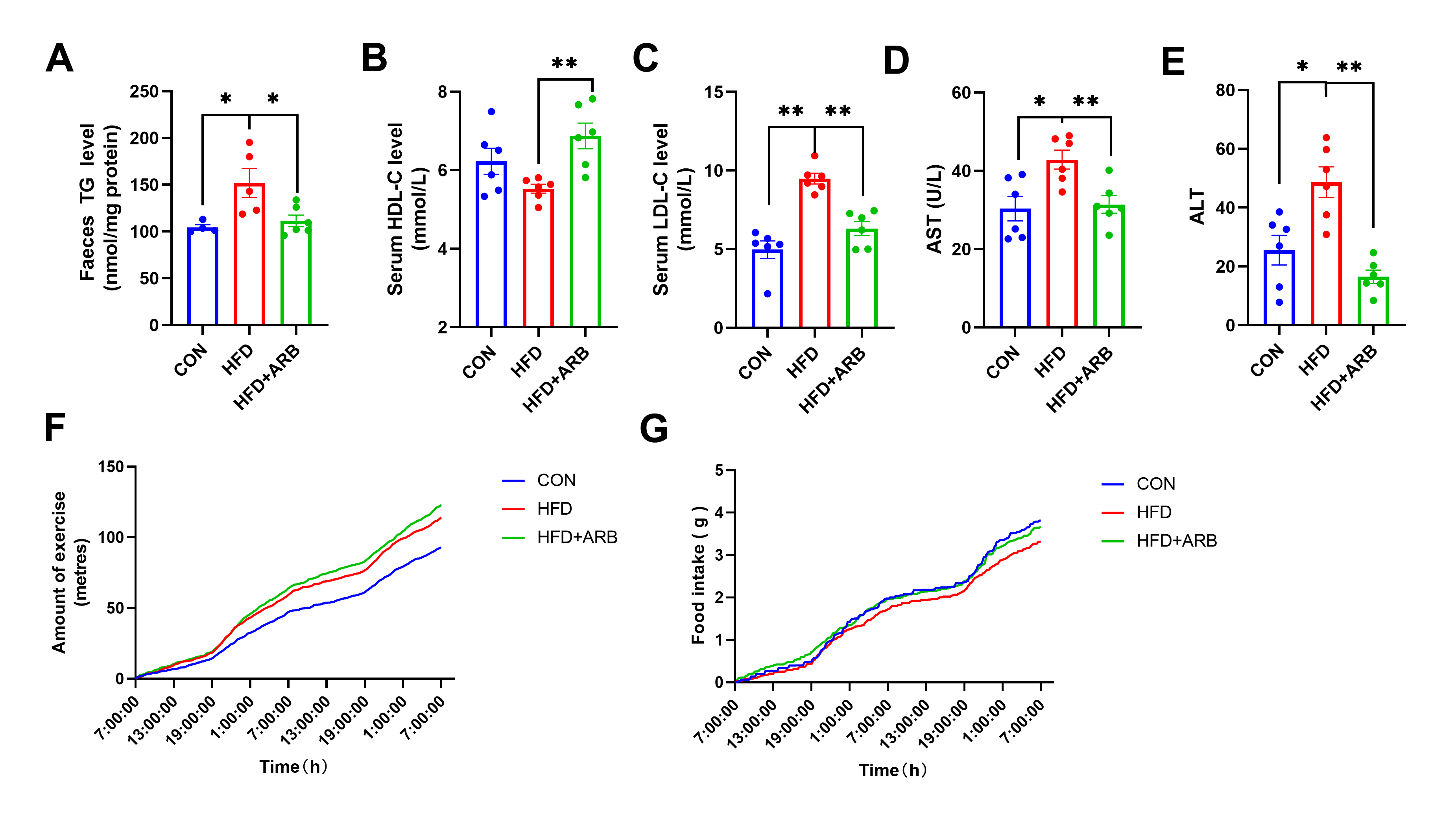


**Figure S3. Effect of ARB on fat deposition and ferroptosis in mice. (**A) Fecal TG content in mice (n=6 per group). (B-E) Serum HDL-C, LDL-C, AST, ALT content in mice (n=6 per group). (F) Exercise level (n=3-4 per group). (G) Food intake (n=3-4 per group). Data are mean ± SEM; One-way ANOVA was used between three or more groups, ∗*P* < 0.05; ∗∗*P* < 0.01.


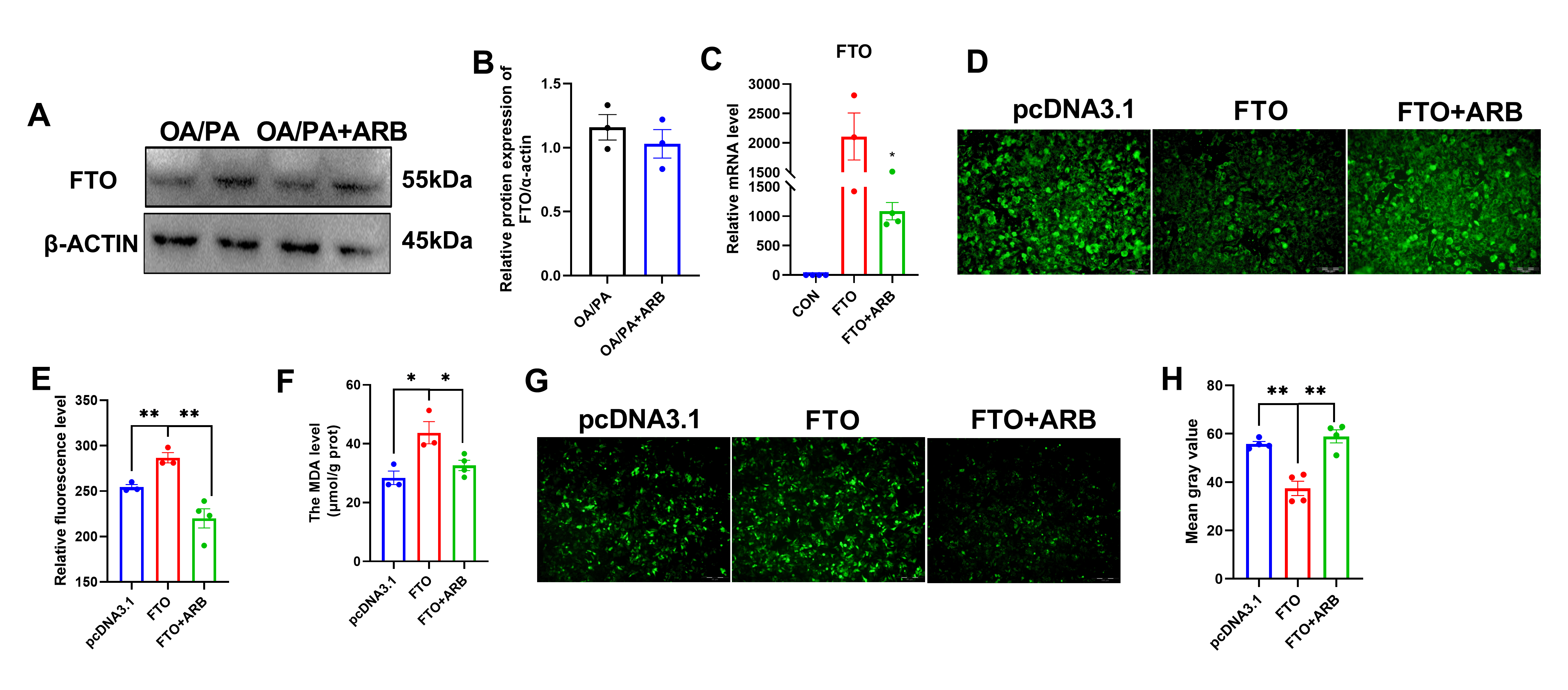


**Figure S4. FTO is the target of ARB action.** (A-B) Protein expression levels of FTO (n=3 per group). (C) qRT-PCR assay of FTO overexpression (n=3-4 per group). (D-E) ROS staining with the DCFH-DA probe (n=3-4 per group). (F) Effect of FTO overexpression on intracellular MDA (n=3 per group). (G-H) Mitochondrial staining (n=4 per group). Data are mean ± SEM, *n* ≥ 3; The data between two groups were analyzed by independent samples t-test, and one-way ANOVA was used between three or more groups, ∗*P* < 0.05; ∗∗*P* < 0.01.


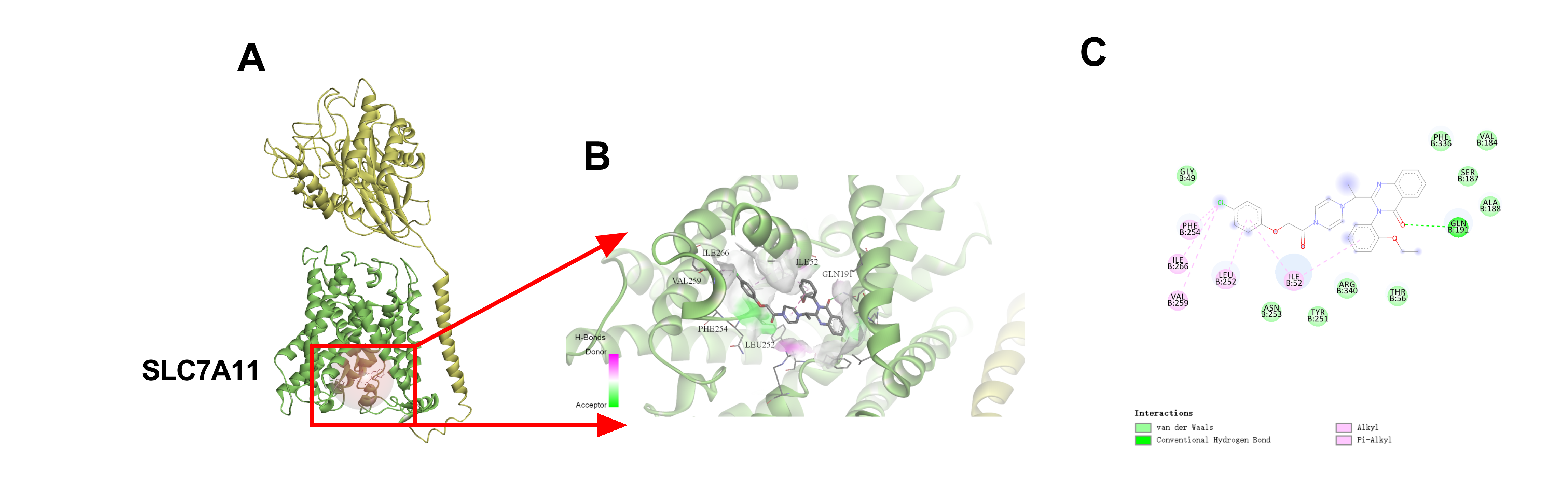


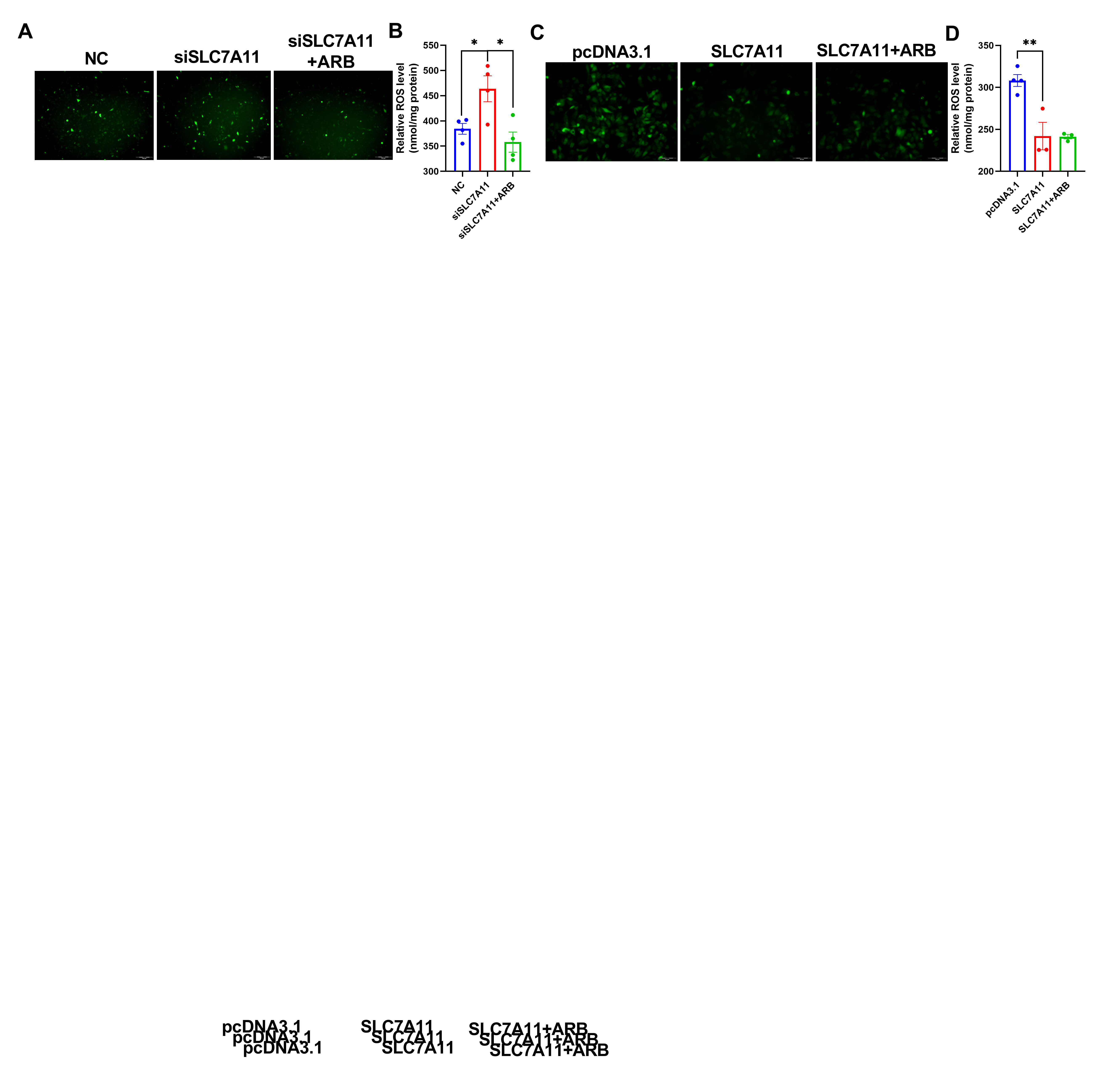
**Figure S5. Erastin is a specific inhibitor of SLC7A11.** (A-C) Molecular docking results of Erastin and SLC7A11. The SLC7A11 is denoted in blue, Erastin is denoted in gray and green circles represent hydrogen, light green circles represent hydrophobic force, purple circles represent van der Waals power.

**Figure S6. Effect of SLC7A11 on ROS.** (A-B) Detection of ROS using DCFH-DA probe after knockdown of SLC7A11 (n=4 per group). (C-D) Detection of ROS using DCFH-DA probe after overexpression of SLC7A11 (n=3-4 per group). Data are mean ± SEM, n ≥ 3; One-way ANOVA was used between three or more groups, ∗P < 0.05; ∗∗P < 0.01.


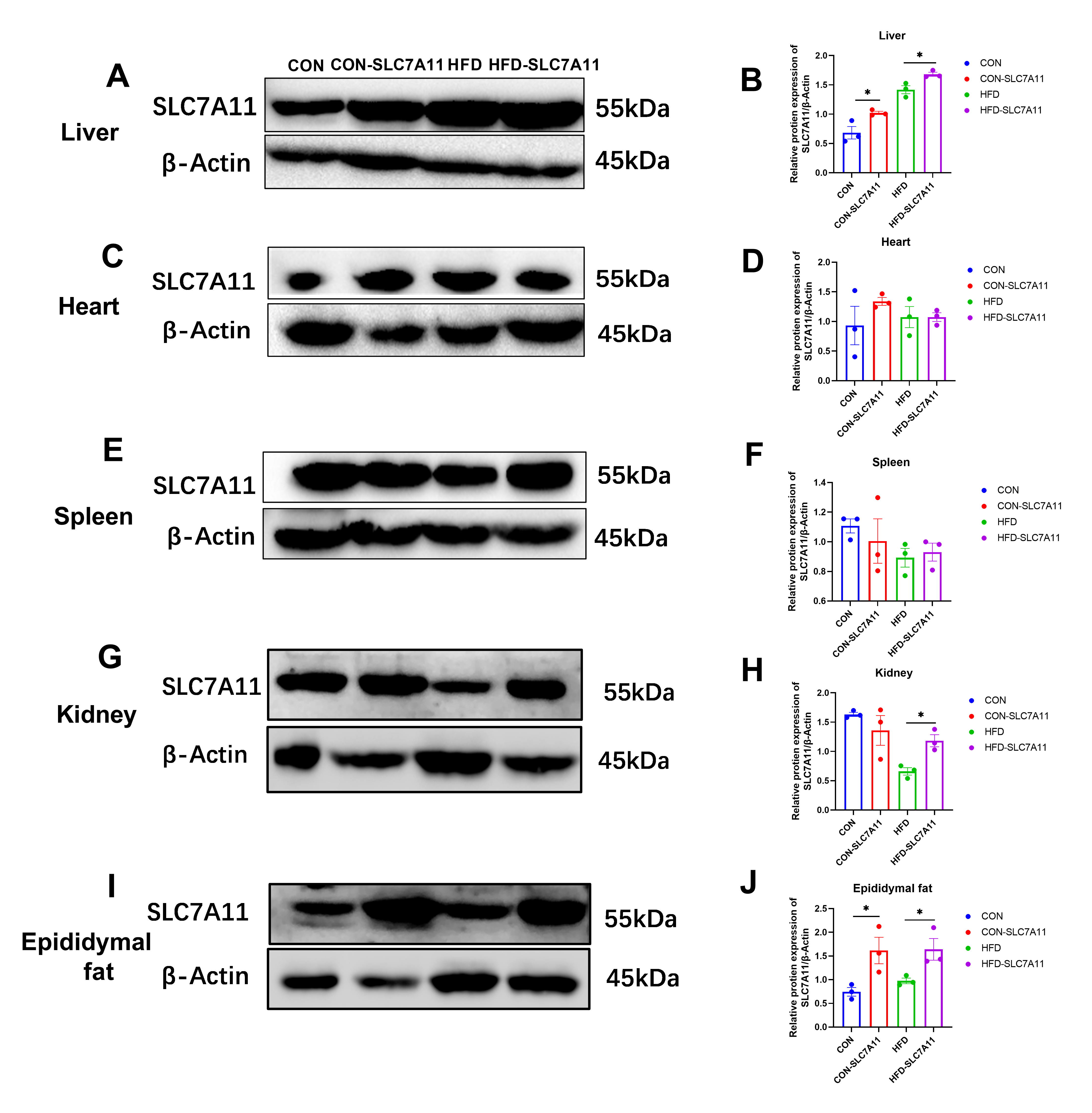


**Figure S7. Tissue expression profiles of SLC7A11 adenovirus-infected liver mice.** (A-B) Liver. (C-D) Heart. (E-F) Spleen. (G-H) Kidney. (I-K) Epididymal fat (n=3 per group). Data are mean ± SEM, *n* = 3; One-way ANOVA was used between three or more groups, ∗*P* < 0.05; ∗∗*P* < 0.01.


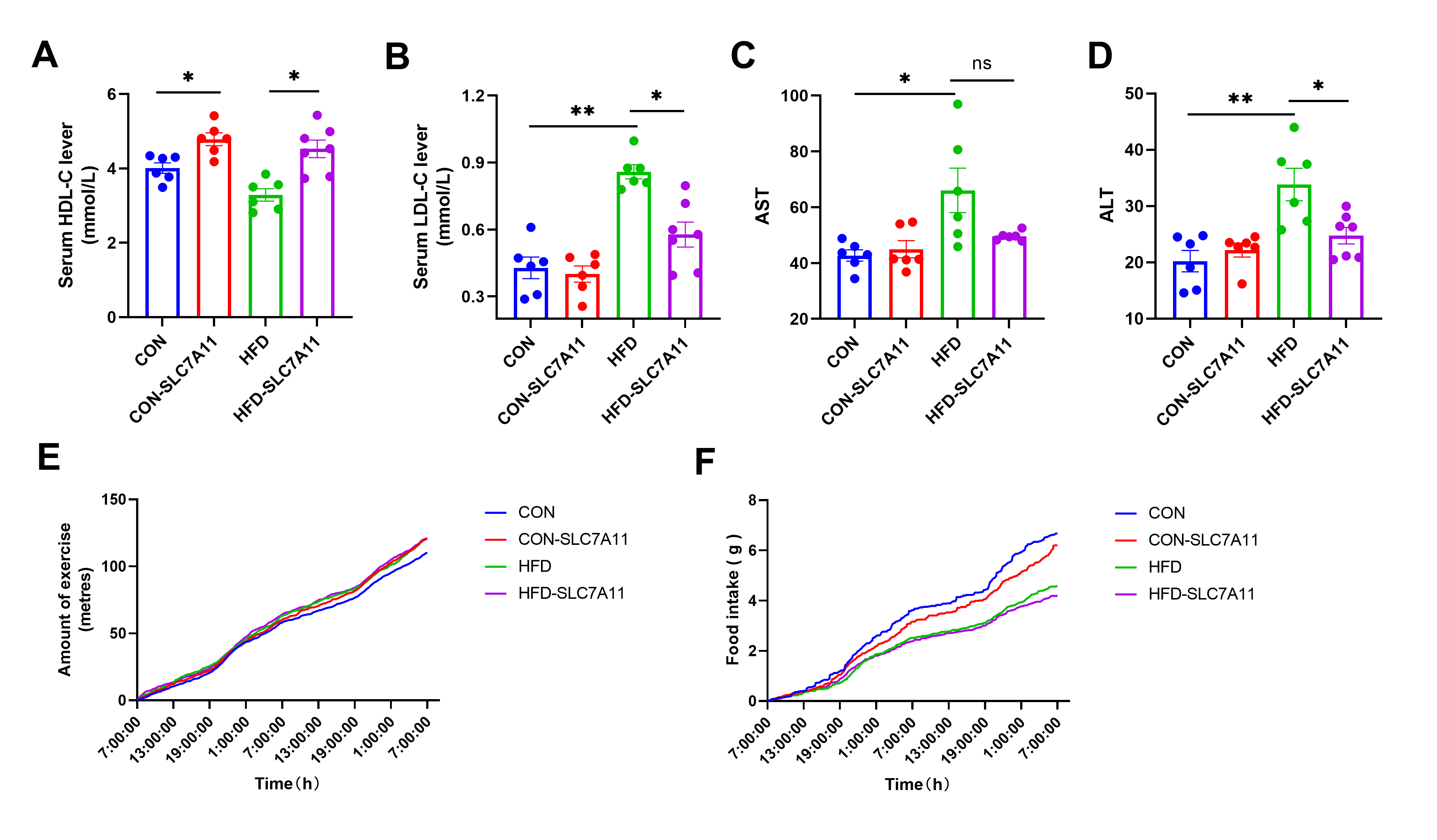


**Figure S8. AAV-mediated SLC7A11 hepatic overexpression alleviates fatty liver energy metabolism in mice.** (A-D) Serum HDL-C, LDL-C, AST, ALT levels in different groups of mice (n=6-7 per group). (E) Exercise level (n=5 per group). (F) Food intake (n=5 per group). Data are mean ± SEM, n ≥ 5; One-way ANOVA was used between four groups, ∗P < 0.05; ∗∗P < 0.01.
